# Lignans in *Schisandra chinensis* green extracts: quantitative analysis and evaluation of tyrosinase inhibitory activity through spectrophotometric assay, *in silico* studies, and STD NMR spectroscopy

**DOI:** 10.3389/fpls.2026.1844111

**Published:** 2026-07-08

**Authors:** Luciana Maria Polcaro, Michela Aliberti, Gabriel Rocha, Jesus Angulo, Giuseppe Bifulco, Sonia Piacente, Gianluigi Lauro, Milena Masullo, Antonietta Cerulli

**Affiliations:** 1 Department of Pharmacy, University of Salerno, Fisciano, SA, Italy; 2 Institute for Chemical Research (IIQ), Consejo Superior de Investigaciones Científicas (CSIC)─University of Seville, Seville, Spain

**Keywords:** anti-tyrosinase activity, *in silico* studies, LC-ESI/QTrap/MS/MS, lignans, *Schisandra chinensis*, STD NMR spectroscopy

## Abstract

The fruits of *S. chinensis* (Turcz.) Baill. (Schisandraceae) are widely used in Traditional Chinese Medicine for their sedative, anti-stress, and cognitive-enhancing properties, which are attributed to the main bioactive constituents belonging to the lignan class. In this work, green extracts of *S. chinensis* fruits were obtained by Solid Liquid Dynamic Extractions-Naviglio (SLDE) and Ultrasound Assisted extractions (UAE) using 100% EtOH, 75% EtOH/H_2_O, and 50% EtOH/H_2_O, and analyzed by LC-MS analysis. Tyrosinase inhibitory activity of isolated compounds was evaluated using a spectrophotometric assay, while Saturation Transfer Difference (STD) NMR experiments and *in silico* studies were conducted to investigate the interaction between the most active lignan, granschisandrin (1), and tyrosinase. A total of 25 lignans were isolated and quantified by LC-ESI/QTrap/MS/MS. The EtOH extract obtained by SLDE-Naviglio showed the highest content of lignan derivatives (0.036–7.626 mg/g extract). Among these lignans, granschisandrin (1) emerged as the most active compound, with an IC_50_ value of 142.75 µM, comparable to that of kojic acid (IC_50_ = 100.07 µM), used as a reference compound. STD NMR and molecular modeling studies confirmed its interaction with tyrosinase and supported that granschisandrin acts as an allosteric inhibitor of the enzyme.

## Introduction

*Schisandra chinensis* (Turcz.) Baill. (Schisandraceae) is one of the most valued medicinal plants widely used in traditional Chinese medicine because it is a rare, multi-functional tonic herb that strengthens vital organs, enhances mental clarity, and promotes balance and longevity (Sun et al., 2021). It is known that *Schisandra* helps the body adapt to physical, mental, and emotional stress; it strengthens resistance to fatigue and is commonly used to support people experiencing chronic stress or weakness (Nowak et al., 2019). Moreover, improves concentration, memory, and alertness and helps with anxiety, restlessness, and poor sleep (Ko and Leung, 2024). In traditional Chinese medicine texts, *Schisandra* is also classified as a superior (longevity) herb; traditionally used to slow aging and increase life force, in fact, it’s often included in tonic formulas for long-term health maintenance (Nowak et al., 2019).

Its fruits are known for their neuroprotective, anti-inflammatory, immunostimulatory, hepatoprotective, and skin-protective properties (Choi et al., 2006; Fu et al., 2025).

Lignans are the most abundant constituents of *S. chinensis* fruits (MacRae and Towers, 1984; Zhang et al., 2012). Chemically, they are phenylpropane dimers derived from the shikimic acid pathway, in which the individual units are joined via their side chains (Berenshtein et al., 2024). Lignans can be classified into eight distinct subgroups based on the cyclization of the molecule and the manner in which oxygen is incorporated into the skeleton. These eight subgroups are: furofuran (FF), furan (FR), dibenzylbutane (DB), dibenzyl-butyrolactone (DBL), aryltetraline (AT), arylnaphthalene (AN), dibenzocyclooctadiene (DCO), and dibenzylbutyrolactol (DBLL) (Umezawa, 2003; Li et al., 2013). Lignans have received considerable attention in the literature from both chemists and pharmacologists over the past few decades due to their intriguing structures and potent biological activities, including their notable anti-neuroinflammatory effects in Parkinson’s disease (Wang et al., 2024b). In our previous manuscript, *S. chinensis* fruits were extracted by decoction, maceration, ultrasound-assisted (UAE), and dynamic solid-liquid (SLDE-Naviglio) extraction using EtOH and EtOH/H_2_O mixtures. The chemical profiles of the extracts were established by a combined approach of LC-HR-MS and NMR analysis, and the multivariate statistical analysis of these data showed how the extracts obtained by UAE and SLDE were the richest ones in lignan derivatives (Polcaro et al., 2024b). Therefore, the fruits of *S. chinensis* were subjected to an SLDE extraction technique using 50% EtOH/H_2_O, and the extract was purified to afford 25 lignans belonging to four of the eight lignan subclasses (FR, DB, AT, and DCO). Quantitative analysis of lignans was performed. Moreover, to highlight the occurrence of bioactive lignans, the tyrosinase inhibitory activity of all 25 lignans was evaluated by spectrophotometric assay. Once a furan lignan, granschisandrin (**1**), was identified as the most active compound, further investigations into the nature of the binding with tyrosinase were conducted using STD NMR and *in silico* studies.

## Material and methods

### Extraction, isolation, and characterization of the lignans

The fruits were extracted using the UAE and SLDE-Naviglio techniques. 100% EtOH, 75% EtOH/H_2_O, and 50% EtOH/H_2_O were used for each extraction procedure as previously reported in Polcaro et al., 2024a (Polcaro et al., 2024b). The extract of *S. chinensis* fruits obtained by SLDE-Naviglio, using 50% EtOH/H_2_O, was first subjected to repartition with n-BuOH/H_2_O (1:1) to remove free sugars (Masullo et al., 2022). Successively, the butanol fraction (3g) was submitted to size-exclusion chromatography using a Sephadex LH-20 column (25–100 mm Pharmacia). A total of 64 fractions were obtained, monitored by TLC (Thin Layer Chromatography), and analysed by an RP-HPLC-UV system (Agilent Technologies 1260 Infinity), setting double wavelength at 254 nm and 290 nm. Fractions were dissolved in MeOH in a concentration of 10 mg/100 μL. The elution gradient was obtained using water with 0.1% formic acid as eluent A and acetonitrile with 0.1% formic acid as B at a flow rate of 2.0 mL/min. A Phenomenex Sinergy Luna 5µm C18 column (250 × 10 mm) was used. The HPLC gradient started at 5% B and increased to 80% in 30 min; after 10 min % B was at 100%, and it was held at 100% for 5 min before returning to the starting percentage. The fractions were submitted to HPLC, and the isolated compounds, with the indication of retention times and amount, are reported in **Supplementary Material** (**Supplementary Table 1**). Subsequently, the structures of the isolated compounds were unambiguously elucidated by 1D- (^1^H and ^13^C) and 2D- (HSQC, HMBC, and COSY) NMR experiments. The purity of these compounds (>99%) was determined by HPLC analysis.

### Quantification of lignans in *S. chinensis* extracts

Quantitative analyses were performed using an LC-ESI/QTrap/MS/MS system operating in Multiple Reaction Monitoring (MRM) mode. The instrument was operated in positive ionization mode. Chromatographic separation was achieved on a Luna Omega C18 column (100 mm × 2.1 mm i.d.) at a flow rate of 0.3 µL/min. The mobile phase consisted of water with 0.1% formic acid (A) and acetonitrile with 0.1% formic acid (B), both v/v. The gradient program was as follows: 5% B held for 50 s, increased to 81% B in 1.0 min and maintained for 4.0 min, then increased to 95% B in 1.0 min and held for 1.0 min, before returning to initial conditions in 1.5 min. Stock solutions (1 mg/mL) of isolated compounds used as external standards (ES) were prepared in methanol and subsequently diluted to obtain eight working concentrations (0.001–10 µg/mL). To each standard solution and to *S. chinensis* extracts, an appropriate amount of internal standard (IS; cyanidin-3-*O*-β-glucopyranoside) was added to achieve a final concentration of 1 µg/mL. Calibration curves were constructed by injecting 5 µL of each standard solution in triplicate. The ratios of the peak areas of the analytes (ES) to those of the IS were plotted against the corresponding concentrations and processed using weighted linear regression. Data acquisition and quantification were performed using Analyst 1.6.2 software (AB Sciex).

Method validation was conducted in accordance with the European Medicines Agency (EMA) guidelines for bioanalytical method validation, evaluating linearity, precision, specificity, limit of detection (LOD), and limit of quantification (LOQ). Linearity was assessed through the calibration curves described (**Supplementary Table 3**). The LOQ was determined as the lowest concentration providing a signal-to-noise ratio of 10. At the same time, the LOD corresponded to a signal-to-noise ratio of 3 obtained by serial dilution of standard solutions (**Supplementary Table 3**).

Specificity was confirmed by verifying the absence of interfering signals at the retention times of the target analytes. Matrix effects were evaluated using the internal standard to compensate for potential ion suppression or enhancement, with quantification based on analyte-to-IS response ratios to minimize matrix-related variability and improve analytical accuracy and precision.

Precision was assessed through intra-day (triplicate) and inter-day (three consecutive days) analyses under identical experimental conditions, demonstrating consistent performance and confirming method reliability. Recovery was evaluated by spiking known amounts of standards at low, medium, and high concentration levels into the extract matrix prior to extraction and analysis. Recovery values ranged from 91% to 106%, indicating satisfactory extraction efficiency.

Finally, robustness was tested by introducing small deliberate variations in key analytical parameters, with no significant effect on analytical performance, confirming method stability (Cerulli et al., 2022).

### Evaluation of tyrosinase inhibitory activity

The assay was performed using a 96-well microplate. 30 microliters of the sample (final concentrations of 50, 100, 150, 200, 250, 300 µM) and 50 µL of 100 U/mL mushroom tyrosinase (*Agaricus bisporus*) were treated in 96-well plates and incubated at 37 °C for 15 min. Subsequently, 50 µL of 1 mM L-tyrosine, used as substrate, was added and then reacted at 37 °C for 15 min (Polcaro et al., 2024a). The amount of dopachrome formed was measured at 495 nm using Thermo Scientific™ Multiskan SkyHigh Microplate Spectrophotometer. Solutions of L-tyrosine, phosphate buffer (pH 6.8), and enzyme were prepared in Milli-Q water. Each sample has been tested in triplicate, and the tyrosinase inhibitory activity was calculated using the following equation:


$$tyrosinase\;inhibition\;\left(\%\right)\;=\;\left[1\;-\;\left(S\;-\;S_{0}\right)/\left(C\;-\;C_{0}\right)\right]\;\times \;100$$

where S is the absorbance of the sample, tyrosinase, and L-tyrosine; S_0_ is the absorbance of the sample and L-tyrosine; C is the absorbance of tyrosinase and L-tyrosine, and C_0_ is the absorbance of L-tyrosine. Kojic acid, a known tyrosinase inhibitor, was used as a positive control. IC_50_ values were calculated through non-linear regression and expressed as means ± SD (standard deviation). They were considered statistically significant with values of *p* < 0.05.

### Interaction studies by NMR, sample preparation

Tyrosinase was purchased from Sigma Aldrich (isolated from the mushroom species *Agaricus bisporus*). Granschisandrin (1) was isolated from the 50% EtOH/H_2_O SLDE-Naviglio extract of *S. chinensis* fruits.

For binding studies, tyrosinase and granschisandrin (1) concentrations of 16.5 µM and 1 mM, respectively, were used. Tyrosinase was prepared in 50 mM phosphate buffer, pH 6.8 in D_2_O. For the STD NMR competition experiment, kojic acid was purchased from Sigma-Aldrich and prepared at a concentration of 1 mM.

### Interaction studies by NMR, STD NMR experiments

For the characterization of compound 1, ^1^H NMR (**Supplementary Figure 1**), HSQC, HMBC (10–160 ppm spectral width), and ROESY spectra were acquired (**Supplementary Figures 2**-**4**). In all experiments D_2_O/DMSO−d6 (95%/5% v/v) mixture was used as solvent. STD NMR experiments for tyrosinase binding to granschisandrin were carried out at 25 °C using a 600 MHz spectrometer equipped with an UltraShieldPlus superconducting magnet with a high-performance cryoprobe optimized for ^1^H, ^13^C, ^15^N and ^19^F detection. The Reduced-Dataset (rd-STD NMR) approach was used, with 0.75 and 6 seconds as short and long saturation times.

For STD NMR experiments, *stddiff.3* pulse program was used with 25 ms of spin-lock time to remove the residual protein signals, 40 dB of power of a shaped pulse to irradiate the protein, and -1/40 ppm as irradiation frequency for on/off resonance spectra. STD NMR competition experiments were also performed using the same parameters described above for the STD NMR experiments. Analysis was performed by adding the Kojic acid to the ligand/enzyme solution.

### *In silico* studies

The 3D structure of *Agaricus bisporus* tyrosinase protein (PDB ID: 2Y9X (Ismaya et al., 2011)) was downloaded by Protein Data Bank and prepared using Protein Preparation Wizard tool (Madhavi Sastry et al., 2013; Schrödinger Release 2025-2; Protein Preparation Workflow; Epik): hydrogen atoms were added, and bond orders were assigned. As reported in the Results and Discussion section, molecular docking experiments were performed on the catalytic and an allosteric site of the protein. In detail, prior to proceeding with molecular docking experiments with kojic acid, which is known to bind the catalytic site, the grid box was generated using the co-crystallized ligand tropolone as a reference compound (PDB ID: 2Y9X (Ismaya et al., 2011)). The coordinates of the obtained grid were X = -6.62 Å, Y = -23.44 Å, Z = -35.58 Å, to cover the catalytic site of the enzyme. The inner and outer box dimensions of the grid were 30 × 30 × 30 Å and 50 × 50 × 50 Å, respectively. To validate the grid box and docking protocol, a redocking experiment was performed using Glide software (Schrödinger Suite 2025-2) in the Extra Precision (XP) mode to reproduce the binding mode of tropolone reported in the crystal structure. The generated molecular docking pose successfully reproduced the experimental binding mode observed for the co-crystallized ligand, yielding an in-place all-heavy atom root mean square deviation (RMSD) of 0.48 A. (**Supplementary Figure 7**).

For the molecular docking experiments targeting the allosteric site of tyrosinase, an additional grid box was generated by selecting the following amino acid residues to define the binding region: Gly149, Ala212, Ile217, Phe224. The coordinates of the obtained grid were X = 4.38 Å, Y = -34.68 Å, Z = -38.58 Å. The dimensions of the inner and outer boxes were 20 × 20 × 20 Å and 50 × 50 × 50 Å, respectively.

Glide software (Yang et al., 2021; Friesner et al., 2006; Halgren et al., 2004; Friesner et al., 2004; Iannitelli et al., 2023; Schrödinger Release 2025-2: Glide, Schrödinger, LLC, New York, NY, 2025) was used for molecular docking experiments, setting Extra Precision (XP) mode: 100,000 poses were generated in the initial docking phase, 800 conformations were retained and subjected to the minimization step. Finally, a maximum of 30 poses were saved for subsequent qualitative and quantitative analysis.

Molecular dynamics simulations were performed using Desmond software (Bowers et al., 2006; Hasegawa, 2010) Schrödinger Release 2025-2: Desmond Molecular Dynamics System). Complexes under investigation (e.g., tyrosinase-kojic acid, tyrosinase-compound 1, tyrosinase with kojic acid and compound 1) were prepared by System Builder using an orthorhombic box with a 10 Å buffer distance and the TIP3P water model for solvation. Also, Na^+^ ions were added to neutralize the system. Furthermore, Na^+^ and Cl^-^ ions were added at a concentration of 0.15 M. The system was subjected to minimization and relaxation at temperatures of 100 K following these steps: 1) NVT Brownian Dynamics (6250 ps); solute non-hydrogen atoms were restrained; 2) NVT simulation (750 ps); with the Langevin thermostat and fast temperature relaxation constant, a velocity resampling every 1 ps and non-hydrogen solute atoms restrained; 3) NPT simulation (750 ps); the Langevin thermostat and barostat were employed, a pressure of 1 atm was set, with slow pressure relaxation constant, fast temperature relaxation constant, velocity resampling every 1 ps, non-hydrogen solute atoms restrained; 4) NPT ensemble simulation (750 ps); non-hydrogen solute atoms restrained; the Langevin thermostat and barostat were employed with dynamically assigned temperature and pressure values (defaulted to 100 K and 1 atm) 5) NPT simulation (1500 ps); the Langevin thermostat and barostat were employed, with fast temperature relaxation constant and a normal pressure relaxation constant. The same protocol was subsequently reiterated, increasing the temperatures to 200 K and then 300 K to gradually stabilize the systems at the final temperature of the MD simulation (300 K).

After that, productive MD simulations of 1 μs were then performed at 300 K; a recording interval of 1.2 ps and an NPT ensemble class (1.01 bar) were set, with 2.0 fs as integration timestep.

The analysis of the MD simulations was performed with the Simulation Interaction Diagram tool (Bowers et al., 2006; Hasegawa, 2010; Schrödinger Release 2025-2: Desmond Molecular Dynamics System). RMSD values for both the protein and ligands were calculated by superimposing each trajectory frame, extracted every 2000 ps, onto the initial structure (frame 0), considering the backbone atoms for the protein and all heavy atoms for the ligand.

### Tyrosinase inhibition kinetic analysis

Kinetic analysis of tyrosinase enzyme for granschisandrin (**1**) was determined using the Lineweaver–Burk plot. Granschisandrin (1) was tested at its IC_50_ concentration by varying the concentration of L-tyrosine as the substrate (0.10, 0.25, 0.50, and 1.00 mM). At 37 °C, the enzyme inhibition reaction was recorded by measuring the absorbance of the microplate reader at 495 nm for 0, 3, 6, 9, 12, and 15 min. The data obtained were plotted as 1/change concentration of product (1/V) against 1/substrate concentration (1/[S]), and Km were calculated. All experiments were performed in triplicate (Fenoll et al., 2003; Athipornchai et al., 2021).

## Results and discussion

### Isolation and identification of lignans

**Figure 1:**
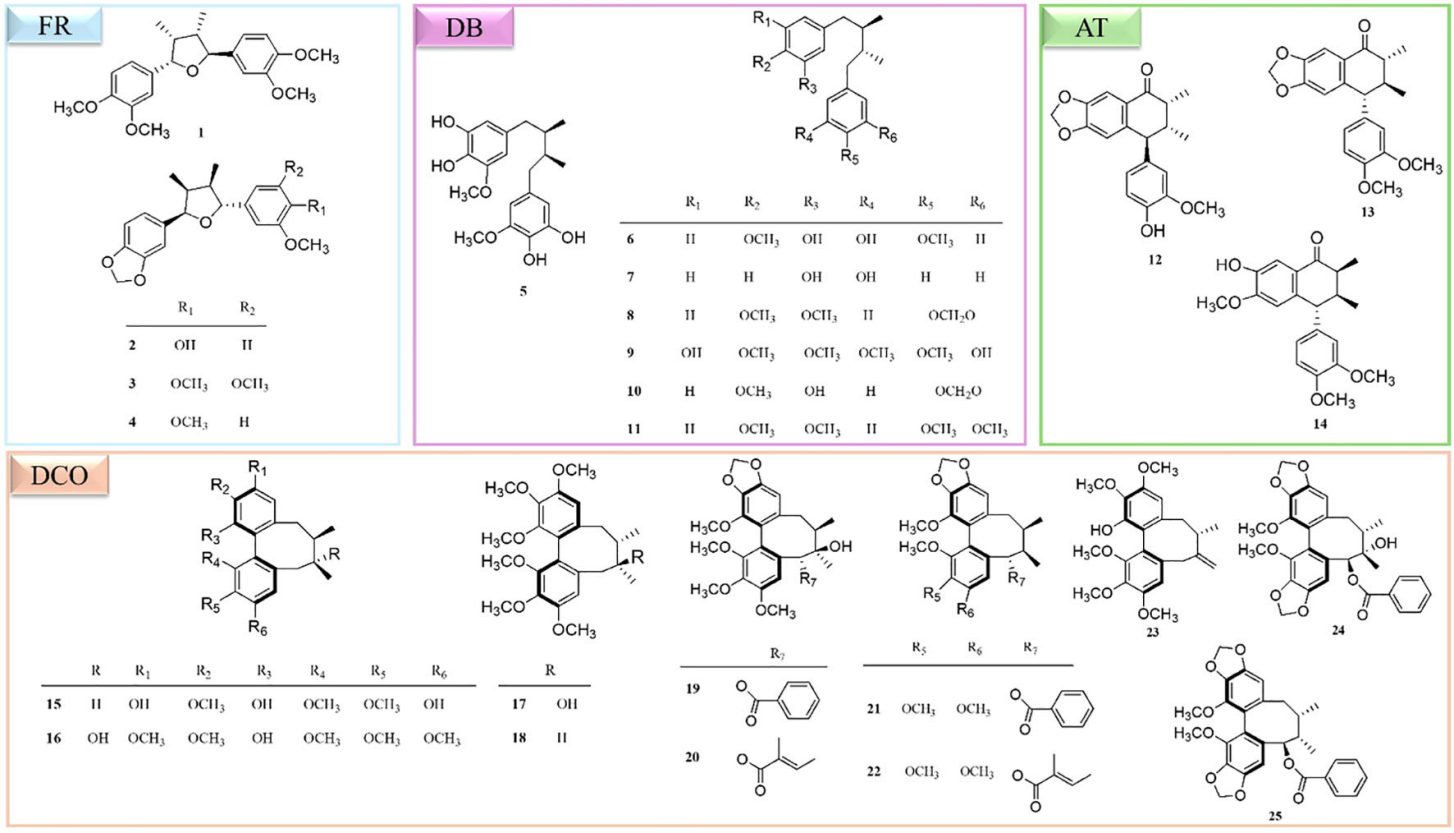
Lignans isolated from *S. chinensis* fruits.

The SLDE-Naviglio extract (50% EtOH/H_2_O) of *S. chinensis* fruits was fractionated on a Sephadex LH-20 column, and afterward, the obtained fractions were further purified by HPLC-UV (**Supplementary Table 1**); in this way, 25 lignans (**Figure 1**) were identified by analysis of their NMR (^1^H and ^13^C NMR, HSQC, HMBC, and COSY experiments) data along with ESIMS and HRMS analysis as previously reported (Polcaro et al., 2024b).

It is possible to group all isolated schisandrin derivatives into different lignan subgroups. In detail, compounds 1–4 belonged to the furan (FR) class, compounds 5–11 belonged to the dibenzylbutane (DB) group, lignans 12–14 belonged to the aryltetraline (AT) type, and finally, lignans 15–25 were representatives of the dibenzocyclooctadiene (DCO) class (Umezawa, 2003).

### Quantitative analysis of lignans in *S. chinensis* extracts

**Figure 2:**
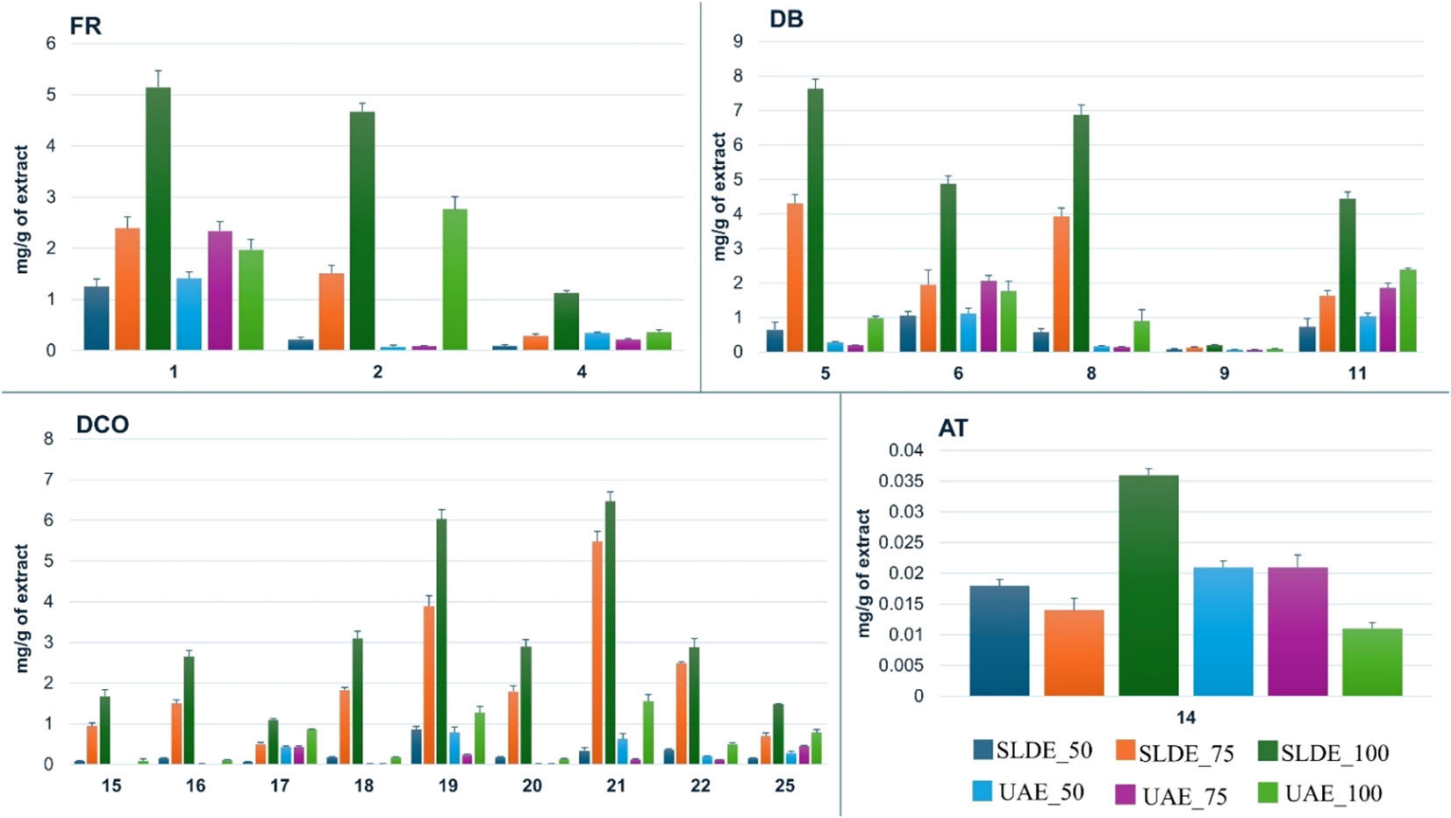
Quantitative analysis of *S. chinensis* isolated lignans.

To date, quantitative analysis in the literature has been reported only for compounds 15, 17–20, and 22 in *S. chinensis* fruits (Sadeghi et al., 2021; Mayer and Meyer, 1999; Walpole et al., 2019). Therefore, herein, the amount (mg/g extracts) of the isolated schisandrin derivatives in *S. chinensis* green extracts (**Figure 2**) was determined.

To quantify schisandrin derivatives (1-25) in *S. chinensis* green extracts, an LC-ESI/QTrap/MS/MS analysis was performed using MRM (Multiple Reaction Monitoring) mode. In detail, MRM is a tandem mass spectrometric technique in which a particular transition from a precursor ion to a product ion is selected for each compound, ensuring a high selectivity and sensitivity (**Supplementary Tables 2**, **3**) (Sadeghi et al., 2021). Considering the fragmentation pattern, the specific MRM transition of each investigated compound was chosen.

The results of the quantitative analyses showed SLDE_100 as the richest extract in schisandrin derivatives (1, 2, 4-6, 8, 9, 11, 14-22, 24, and 25) followed by SLDE_75, with concentration ranges of 0.036–7.626 mg/g extract and 0.014–5.480 mg/g, respectively (**Figure 2**; **Supplementary Tables 4**-**7**).

In detail, among the FR type, compound 1 (5.147 mg/g extract) was the most abundant. Considering the DB subclass of lignans, 5 was the most abundant compound (7.626 mg/g extract). Finally, compound 21 (6.467 mg/g extract) was the most abundant among the lignans belonging to the DCO class (**Figure 2**; **Supplementary Table 6**). The concentrations of compounds 3, 7, 10, 12, 13, 23, and 24 were very low; therefore, they were reported as not detected (N.D.).

### Tyrosinase inhibitory activity of lignans

Tyrosinase is an oxidoreductase enzyme (more precisely a monooxygenase or polyphenol oxidase) containing copper ions (Cu²^+^) in the active site (Rezaei et al., 2018; Qu et al., 2020). This enzyme can be found in animals, plants, and fungi. It is a type 3 copper protein in which the two cupric ions are an integral part of the active site and play a fundamental role in its activity (Guo et al., 2025). In mammals, the tyrosinase enzyme is known for its action in catalyzing the first reactions in the synthesis of melanin, the pigment responsible for the color of skin, hair, and eyes, starting from the amino acid tyrosine (Hridya et al., 2016).

Although tyrosinase is mainly expressed in the skin, it is also present in the brain, where it contributes to dopamine oxidation and neuromelanin formation (Iannitelli et al., 2023). Excessive tyrosinase activity may increase reactive quinones and neuromelanin accumulation in dopaminergic neurons, promoting oxidative stress and apoptotic cell death, processes associated with neurodegenerative disorders such as Parkinson’s disease (Carballo-Carbajal et al., 2019). Therefore, tyrosinase inhibition has also been proposed as a potential strategy to limit neurodegeneration-related oxidative damage (Asanuma et al., 2003; Qi et al., 2024; Wang et al., 2024a; Yu et al., 2021).

**Figure 3:**
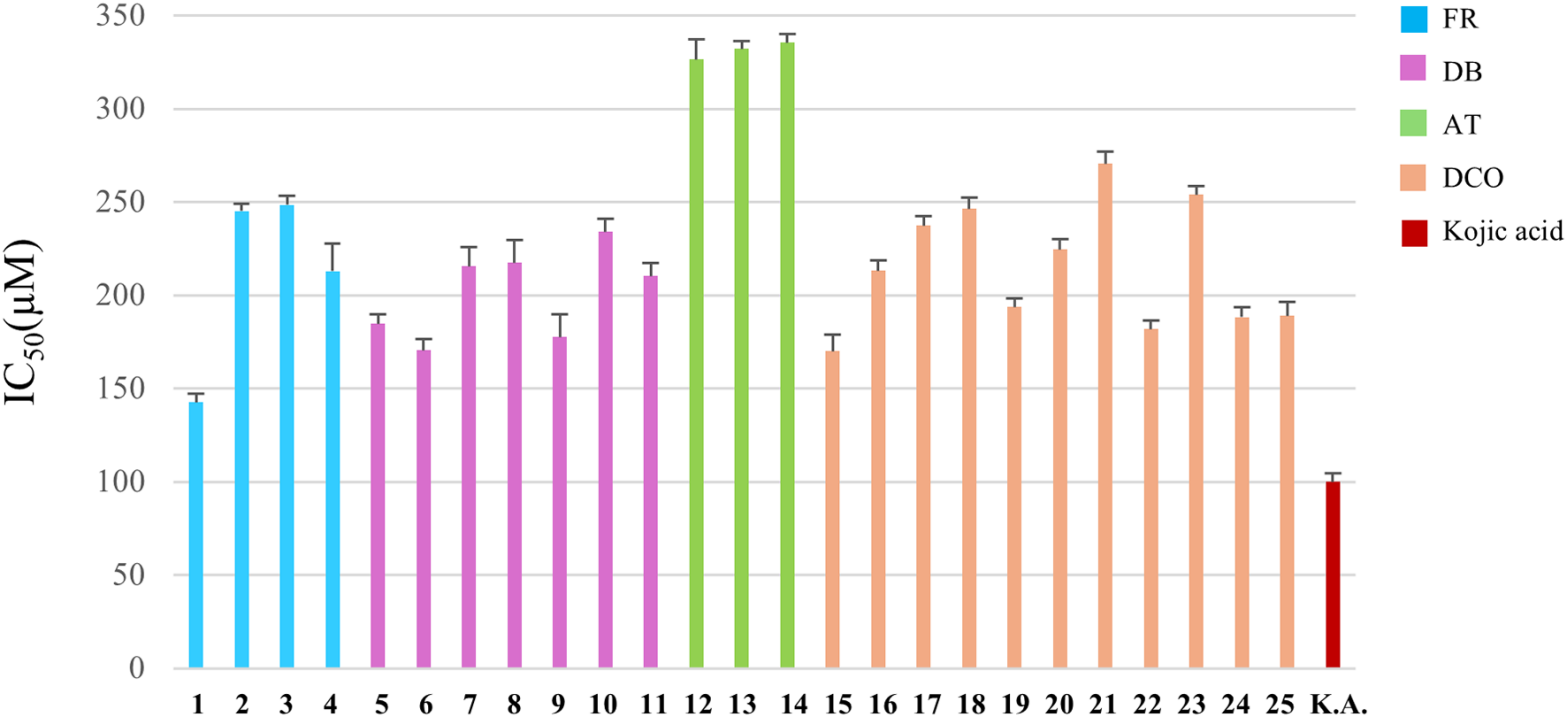
Tyrosinase inhibitory activity of lignans isolated from *S. chinensis* fruits and kojic acid (K.A.).

Tyrosinase inhibitory activity was evaluated using a spectrophotometric assay as a primary screening tool, enabling the rapid assessment of a large number of compounds before progressing to more complex and costly methods. Among available tyrosinase sources, mushroom tyrosinase from *Agaricus bisporus* is commonly used due to its commercial availability and high similarity and homology compared to human tyrosinase (Fenoll et al., 2003; Athipornchai et al., 2021). All 25 isolated compounds, along with kojic acid as a positive control, are shown in **Figure 3** (**Supplementary Table 8**). Some of the tested compounds exhibited activity within the same concentration range as the positive control, such as compounds 1 (granschisandrin), 6, and 15 with IC_50_ values of 142.75, 170.64, and 170.15 µM, respectively. Relative activity (RA) to the positive control (ratio of the IC_50_ inhibitor relative to that of kojic acid under the same assay conditions) was also calculated to compare more accurately the results with previous studies (**Supplementary Table 8**).

**Figure 4:**
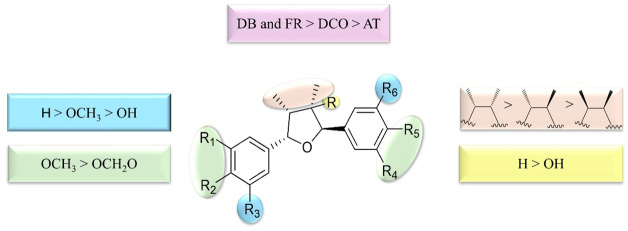
Structure-activity relationships of lignans against tyrosinase.

Considering the high number of molecules tested, a Structure-Activity Relationship (SAR) of lignans against tyrosinase was proposed (**Figure 4**).

In general, FR and the DB lignan-types showed IC_50_ values higher than DCO and AT subclasses; the absence of R increased the activity, and the occurrence of a methoxy or hydroxy group as R_3_ and R_6_ decreased the activity, while the occurrence of methoxy groups as R_1_ and R_2_, or R_4_ and R_5,_ increased the tyrosinase inhibitory activity more than a dioxane cycle.

### STD NMR interaction study

**Figure 5:**
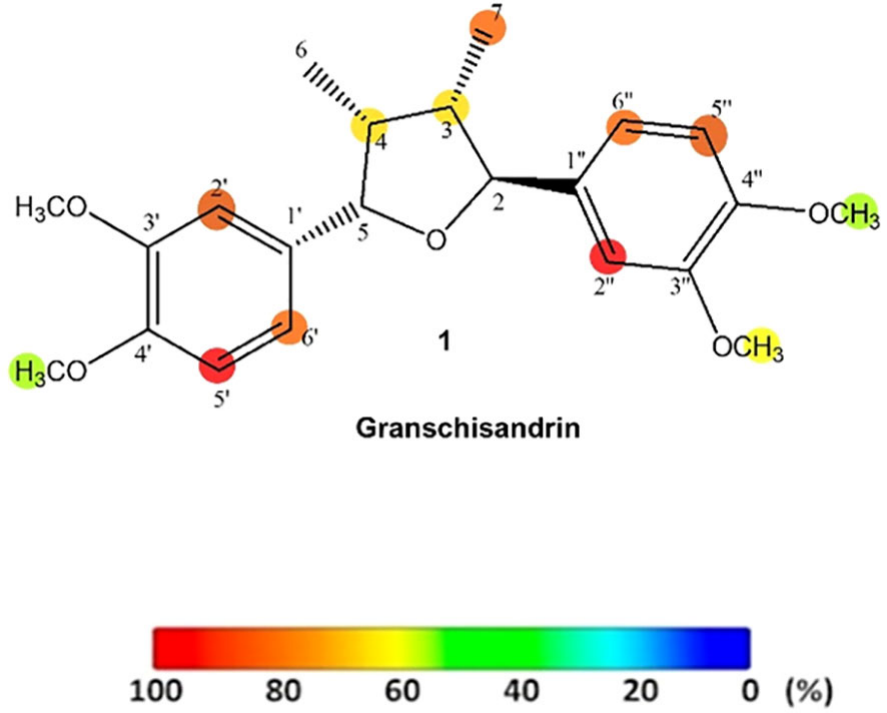
Binding epitope mapping of granschisandrin (1) interacting with tyrosinase from STD NMR spectroscopy.

STD NMR is a powerful technique for studying protein-ligand interactions, enabling the determination of the binding epitope map of the ligand, providing detailed information about the specific contact areas and the affinity of the interactions (Mayer and Meyer, 1999; Walpole et al., 2019; Mayer and Meyer, 2001; Khan et al., 2023). Among all the isolated lignans, 1 (granschisandrin) was selected based on its highest tyrosinase inhibitory activity. To reveal the protons of granschisandrin (1) that were in closer contact with the tyrosinase protein in the binding pocket (binding epitope mapping), an STD NMR experiment was performed. We have used the recently developed reduced dataset (rd-STD NMR) (Rocha et al., 2024) approach, which allows for the determination of accurate binding epitope mappings from initial slopes by using just two saturation times, rather than the traditional time-consuming method that requires constructing a complete build-up curve (Mayer and James, 2004). **Figure 5** shows the binding epitope map of granschisandrin (1); the protons of compound 1 that are in closer contact with the tyrosinase protein in the bound state are colored in red and orange; they are related to the aromatic part and the protons of the methyl group at C-7.

The set of STD NMR integration values at short (0.75 s) and long saturation times (6 s), as well as the key parameters k_sat_, STD_0,_ and the relative STD percentage (epitope) of all the protons of granschisandrin (1), are reported in **Supplementary Table 9**.

**Figure 6:**
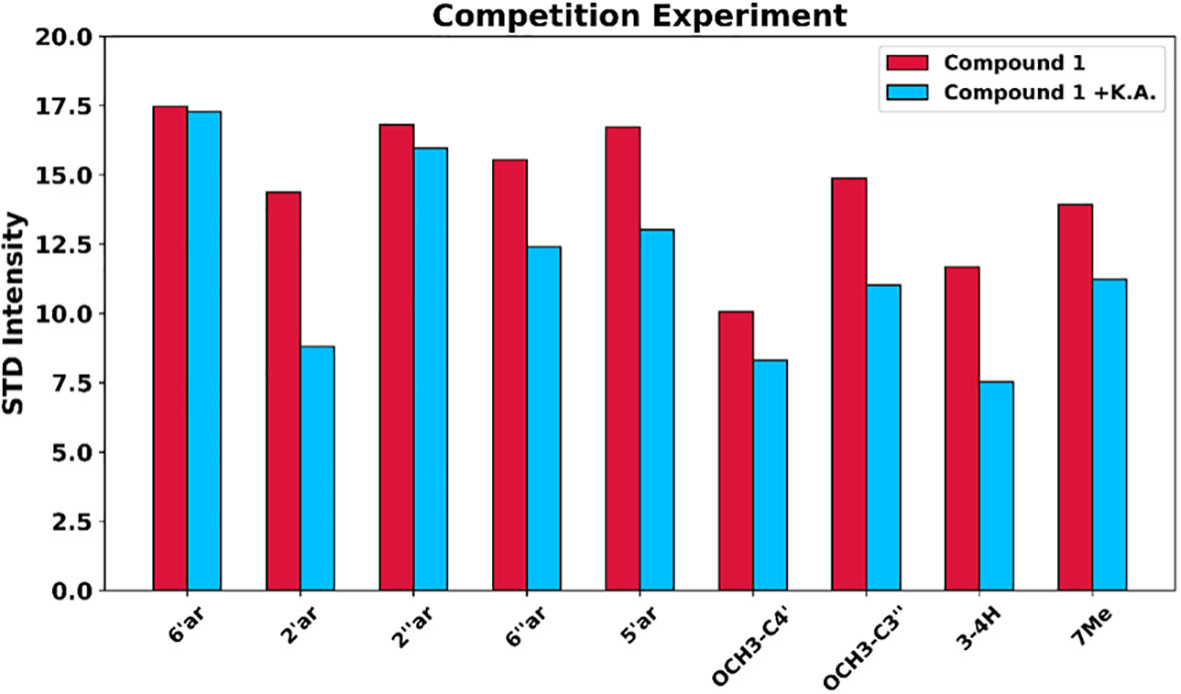
STD NMR competition experiments: STD values of compound 1 in presence of tyrosinase (red) and STD values of compound 1/tyrosinase after addition of an equimolar concentration of kojic acid (blue).

**Figure 7:**
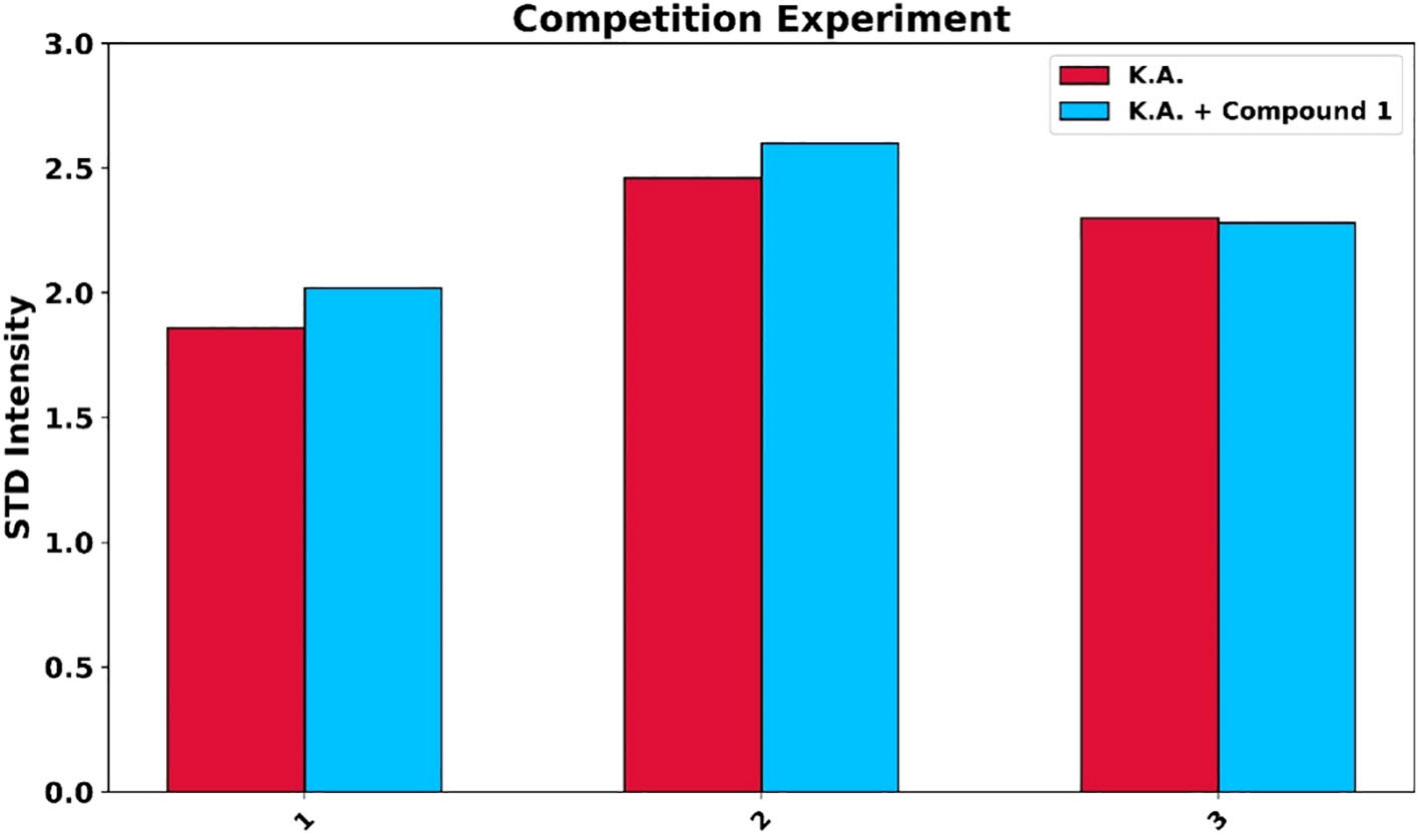
STD NMR competition experiments: STD values of kojic acid in presence of tyrosinase (red) and STD values of kojic acid/tyrosinase after addition of an equimolar concentration of compound 1 (blue).

With the aim of providing further insight into the interaction of 1 with tyrosinase and exploring whether granschisandrin (1) binds the same pocket as kojic acid, a known inhibitor of the enzyme, STD NMR competition experiments were performed between 1 and kojic acid. In the first STD NMR competition experiment, granschisandrin (1) was the first ligand and kojic acid was the second ligand (added in equimolar concentration, **Figure 6**), and in a second STD NMR competition experiment, kojic acid was the first ligand and granschisandrin (1) was the second one (added in equimolar concentration, **Figure 7**).

The results from the first experiment (**Figure 6**) revealed that granschisandrin (1) and kojic acid did not bind the same site of the tyrosinase protein, as no drastic decrease or disappearance of the STD signals of granschisandrin (1) was observed upon addition of kojic acid. Only a slight reduction in the STD values of granschisadrin was observed, which suggested binding to an allosteric site. Indeed, after addition of kojic acid to compound 1, non-linear STD intensity decreases of the protons of compound 1 were observed, suggesting that the two ligands likely bound the tyrosinase protein in two different but close binding sites, possibly sharing binding to some amino acids of the protein, evident by STD intensity decreases at protons 2’ ar, the proton of the methoxy group in position C_4_’ and H3 and H4 of compound 1.

In the second STD NMR competition experiment, after the addition of granschisandrin (1) to the sample of tyrosinase in the presence of kojic acid, no significant changes in the STD intensity of kojic acid were observed (**Figure 7**). In this way, from the two STD NMR competition experiments, it was possible to confirm that the two ligands do not bind the same site of the enzyme.

### *In silico* studies

To corroborate the results obtained from the STD NMR experiments and rationalize the binding mode of granschisandrin (1) at a molecular level, a comprehensive *in silico* investigation was performed using the crystallographic structure of *Agaricus bisporus* tyrosinase (PDB ID: 2Y9X (Ismaya et al., 2011), consistent with the STD NMR experimental setting, see Results and Discussion section), which contains the active site involving two copper ions, and two L subunits with a less defined structural role. In addition, two distinct allosteric sites were reported for this enzyme (Jung et al., 2019). Specifically, the first one is located in proximity to the catalytic site, and it is engaged by phthalic acid, whereas the second site is positioned more distally and it is recognized by the well-characterized inhibitor cinnamic acid. In the present study, as further detailed below, we focused on the first allosteric site in agreement with the binding data arising from the STD NMR experiments.

**Figure 8:**
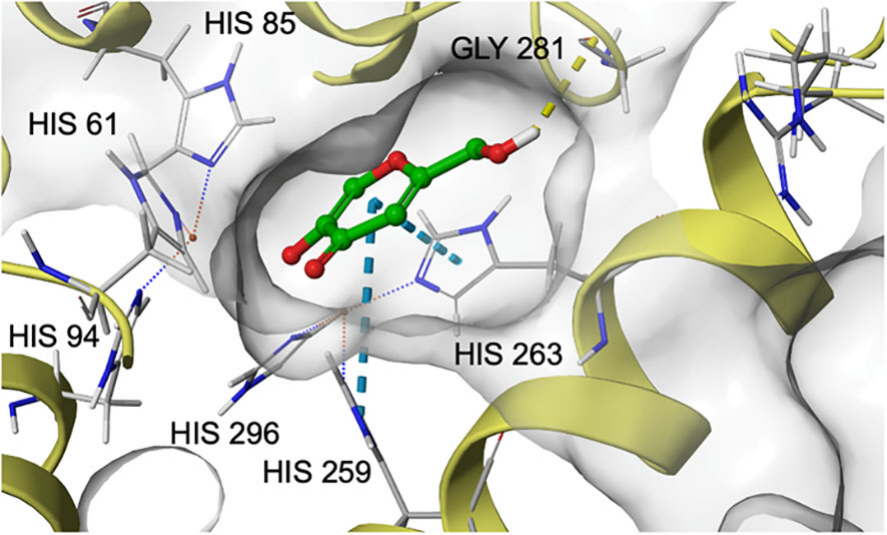
Binding mode of kojic acid (atoms colored by type: carbon in green, oxygen in red, and polar hydrogen in gray) within the tyrosinase binding site (yellow ribbons). π-π stacking interactions and H-bonds are represented as dotted blue and yellow lines, respectively.

**Figure 9:**
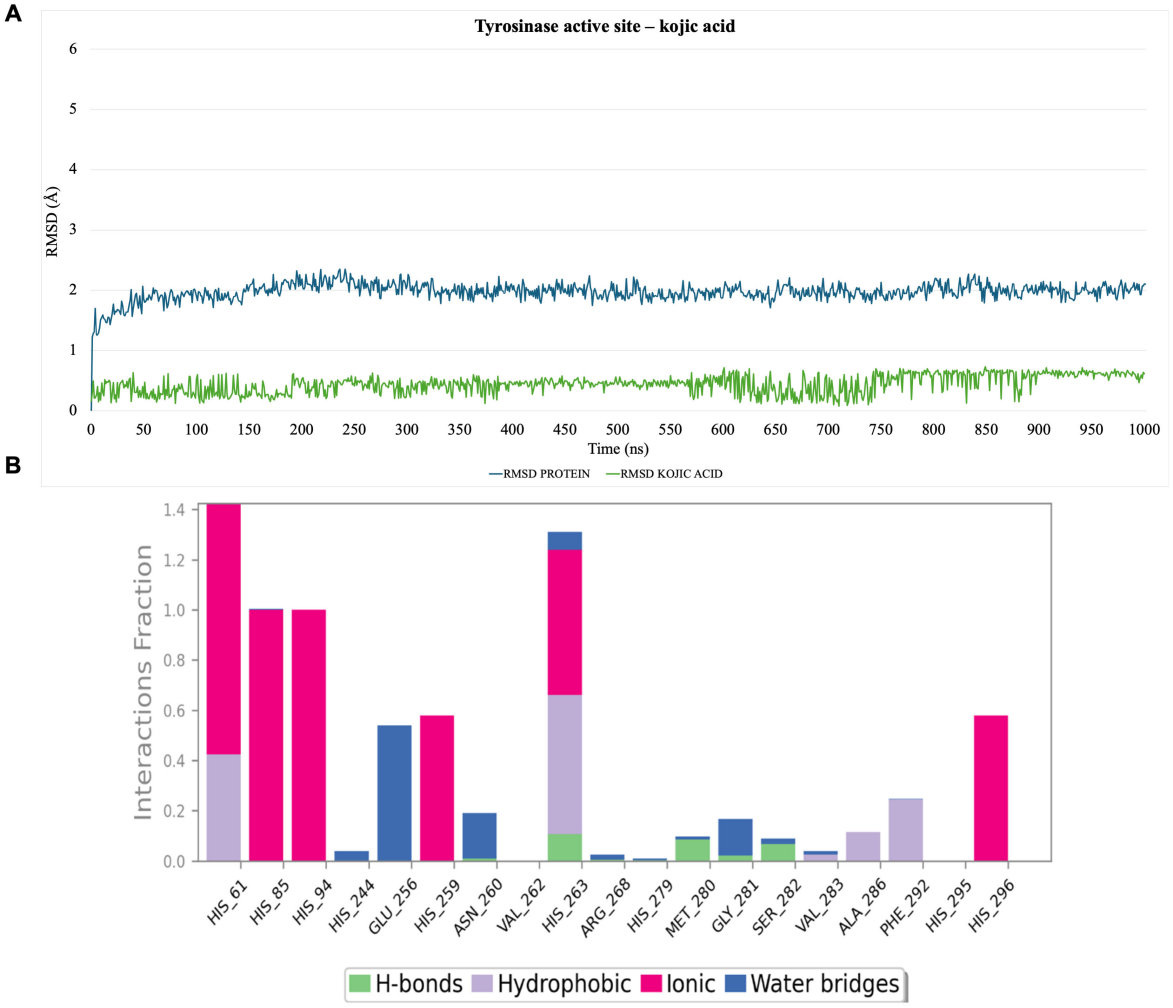
**(A)** RMSD plot of the tyrosinase in complex with kojic acid. Trajectory frame was extracted every 2000 ps and superimposed onto the initial structure (frame 0). RMSD was calculated for backbone atoms of the protein and all heavy atoms of the ligand. **(B)** Tyrosinase – kojic acid interactions monitored throughout the molecular dynamics simulation. Values over 1.0 are possible as some protein residue may make multiple contacts of the same subtype with the ligand.

Initially, we investigated the binding mode of the known inhibitor, kojic acid, at the active site of the enzyme through molecular docking experiments and molecular dynamics (MD) simulations. As illustrated in **Figure 8**, the pyranone core of kojic acid surrounded the region comprising residues His61, His94, His296, His259, and His263, engaging in π-π stacking interactions with His259 and His263. Moreover, the hydroxymethyl group was involved in an H-bond to the Gly281 side chain. This binding mode was further corroborated by a 1 μs molecular dynamics simulation. The root mean square deviation (RMSD) analysis of the trajectory frames indicated a stable behavior of both the protein and the kojic acid throughout the entire simulation. The protein backbone RMSD stabilized around 2 Å after the equilibration phase. In comparison, kojic acid displayed very low RMSD values (< 0.7 Å), suggesting a highly stable binding mode within the tyrosinase active site (**Figure 9A**). Also, the ligand-protein plot showed that kojic acid maintains stable and recurrent interactions with His61 (142%, values over 100% are possible as some protein residue may make multiple contacts of same subtype with the ligand) through both hydrophobic and ionic contacts, with His85 (100%) and His94 (100%) via ionic interactions, and His263 (131%) through a combination of hydrogen bonds, hydrophobic and ionic interactions, as well as water bridges throughout the entire simulation time (**Figure 9B**).

Consistently, the protein root mean square fluctuation (p-RMSF) analysis revealed reduced flexibility for the residues involved in ligand recognition within the catalytic pocket (see Supporting Information; **Supplementary Figure 8**).

**Figure 10:**
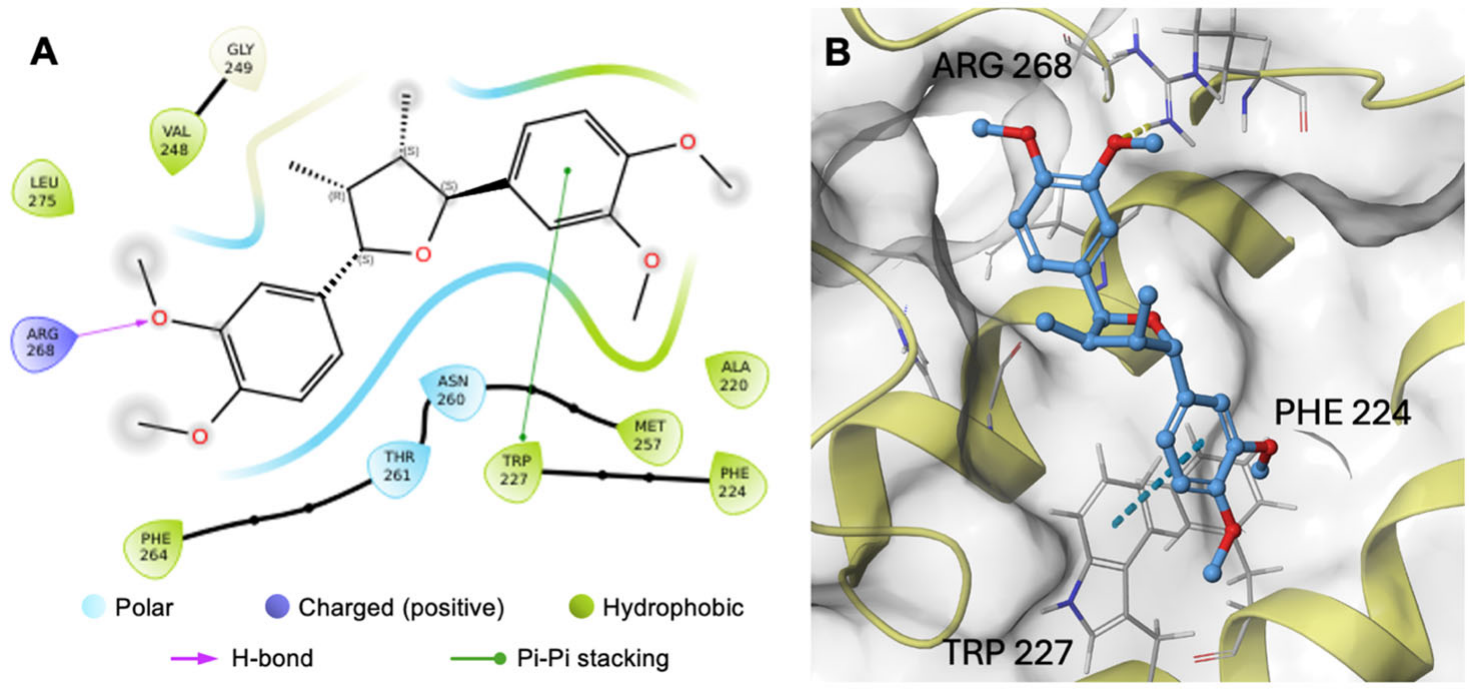
**(A)** 2D representation of compound 1 – tyrosinase interactions. **(B)** Binding mode of compound 1 (atoms colored by type: carbon in light blue, oxygen in red, and polar hydrogen in gray) on the allosteric site of tyrosinase (yellow ribbons). π-π stacking interactions and H-bonds are represented as dotted blue and yellow lines, respectively.

The molecular recognition of compound 1 by tyrosinase, elucidated by STD NMR spectroscopy, revealed that this compound preferentially binds an allosteric pocket located near the catalytic site of the enzyme, where kojic acid is known to bind. Based on these results, molecular docking experiments were performed targeting the adjacent allosteric site, previously identified as the binding site for phthalic acid as a reference inhibitor (Yin et al., 2011; Jung et al., 2019). The docking results were fully consistent with the STD NMR data (**Figure 10A**), showing that compound 1 occupies the adjacent binding pocket by establishing a hydrogen bond with Arg268 and a π-π stacking interaction with Trp227 (**Figure 10B**).

**Figure 11:**
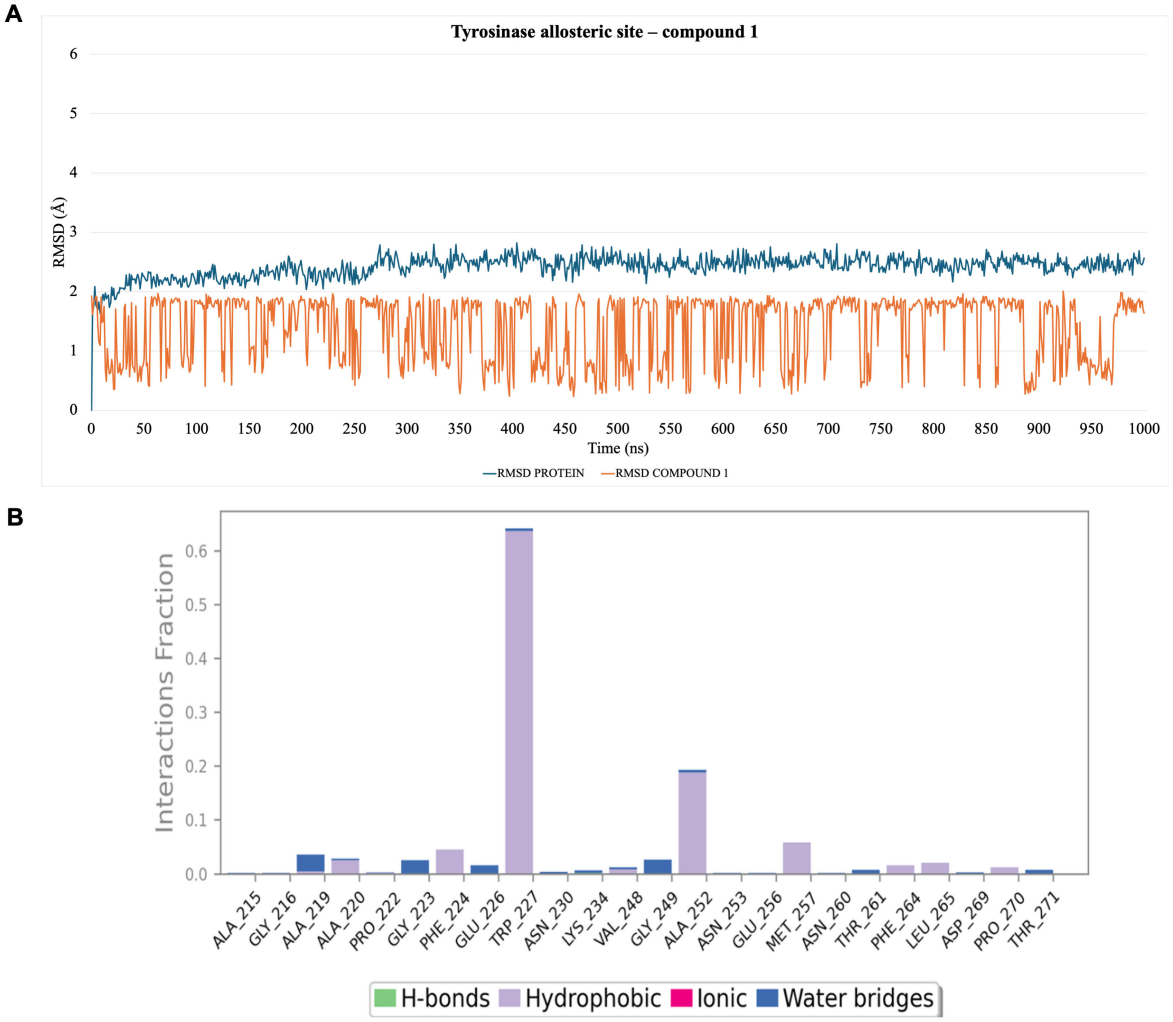
**(A)** RMSD plot of the tyrosinase in complex with compound 1. Trajectory frame was extracted every 2000 ps and superimposed onto the initial structure (frame 0). RMSD was calculated for backbone atoms of the protein and all heavy atoms of the ligand. **(B)** Tyrosinase allosteric site – compound 1 interactions monitored throughout the molecular dynamics simulation. Values over 1.0 are possible as some protein residues may make multiple contacts of the same subtype with the ligand.

This binding mode was also confirmed by a 1 μs molecular dynamics simulation. The RMSD trend showed a structurally stable profile for the protein after the initial equilibration phase, whereas the ligand exhibited fluctuations up to ~1.8 Å, suggesting conformational mobility within the allosteric pocket likely related to ligand flexibility (**Figure 11A**). Despite these oscillations, no complete dissociation event was observed, indicating that compound 1 remained associated with the allosteric region throughout the simulation. This aspect was also corroborated by ligand–protein interaction analysis, which revealed persistent contacts with Trp227 (64%), a key residue of the allosteric site (**Figure 11B**). Moreover, the p-RMSF analysis showed reduced fluctuations for residues involved in ligand recognition within the allosteric cavity, suggesting that this compound contributes to the local stabilization of the binding region (see Supporting Information; **Supplementary Figure 9**).

To further validate the allosteric site as the primary binding region of compound 1, an alternative docking experiment was performed, constraining ligand 1 in the catalytic site. The resulting enzyme-ligand complex was then subjected to a molecular dynamics simulation of 1 μs. Remarkably, in the first few nanoseconds of the trajectory, compound 1 was observed to spontaneously translocate from the catalytic site to the adjacent allosteric pocket. This event was confirmed by the protein-ligand interaction plot, which pointed out interactions with allosteric residues, including Trp227 and Phe224, for most of the simulation time (see Supporting Information; **Supplementary Figure 10**).

**Figure 12:**
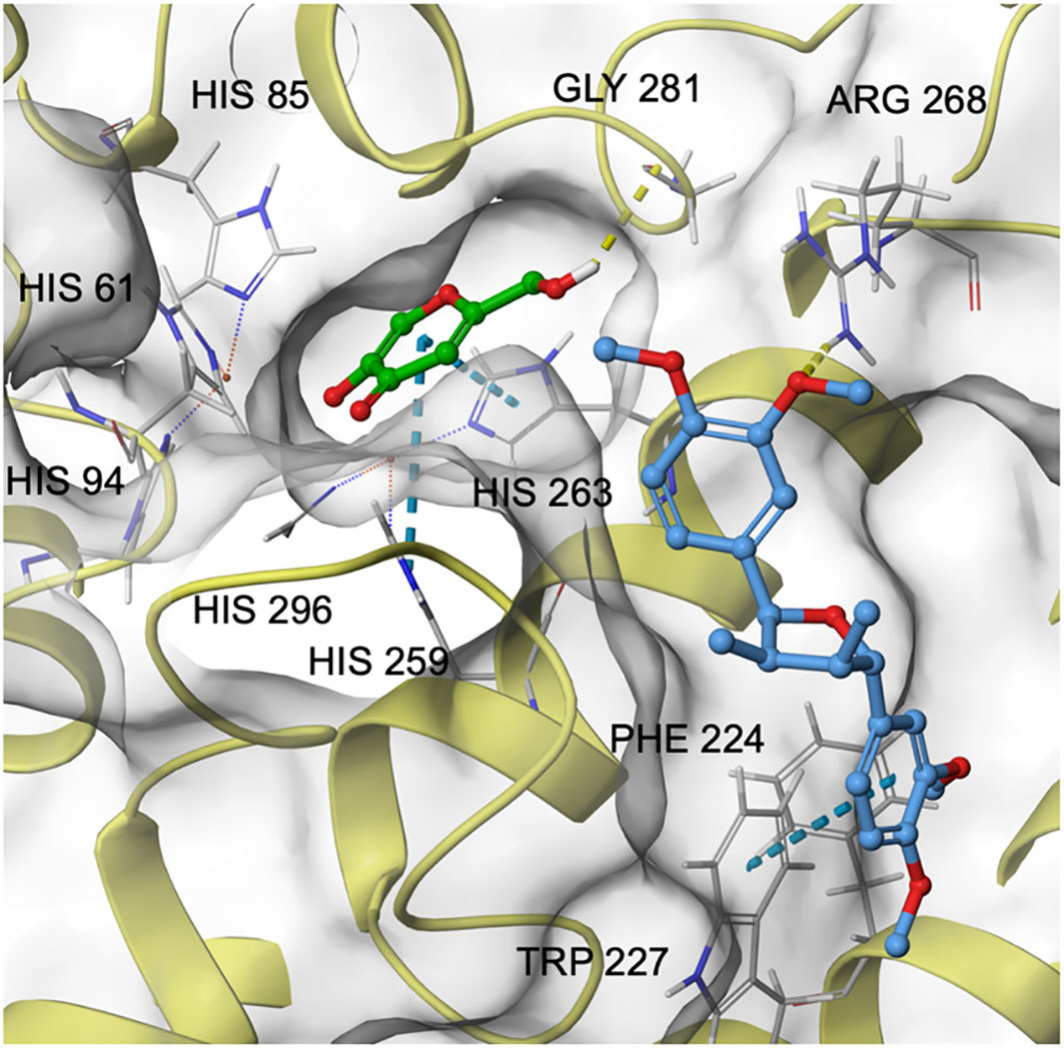
Binding mode of kojic acid (atoms colored by type: carbon in green, oxygen in red, and polar hydrogen in gray) and compound 1 (atoms colored by type: carbon in light blue, oxygen in red, and polar hydrogen in gray) on the catalytic site and allosteric pocket of the tyrosinase (yellow ribbons), respectively. π- π stacking interactions and H-bonds are represented as dotted blue and yellow lines, respectively.

To further support the results of the STD NMR competition experiment, the ligand/protein complex with kojic acid in the catalytic site and compound 1 occupying the adjacent allosteric pocket was reconstructed by molecular docking (**Figure 12**).

To assess the stability of the binding modes and verify that both ligands remained within their respective binding sites, the complex was subjected to molecular dynamics simulation for 1 μs. Throughout the simulation, kojic acid remained stably anchored to the catalytic pocket, maintaining key interactions with the aforementioned residues, particularly His61, His85, and His94, which demonstrated reduced fluctuations (see Supporting Information; **Supplementary Figures 11**, **12**). Similarly, compound 1 preserved its binding mode, interacting with residues belonging to the previously described allosteric region, showing a slight decrease in contact with Trp227 and an increased interaction rate with Phe224 along the simulation (see Supporting Information; **Supplementary Figure 8**), yet globally showing no significant displacement from the proposed allosteric site, supporting the stability of the interaction of 1 in that subsite of the tyrosinase (see Supporting Information; **Supplementary Figure 13**). As expected, the RMSD analysis revealed a behavior consistent with that observed for the individual ligand–protein complexes (see Supporting Information; **Supplementary Figure 9**). The protein rapidly reached equilibrium during the initial phase of the simulation and remained structurally stable throughout the trajectory. Kojic acid displayed consistently low RMSD values over the entire simulation, indicating a stable binding mode within the catalytic site. In contrast, compound 1 exhibited moderate fluctuations, reflecting a certain degree of conformational mobility within the allosteric pocket; however, no dissociation events were observed, suggesting that the ligand remained stably associated with the allosteric binding site during the simulation.

### Kinetic analysis of tyrosinase inhibition

The inhibition mode of granschisandrin (1) against tyrosinase was preliminarily determined using Lineweaver–Burk plots by comparing the kinetic parameters obtained in the presence of granschisandrin (1), tested at its IC_50_ value in triplicate, with those of the control reaction performed in the absence of the inhibitor (**Supplementary Figure 14**). The Lineweaver–Burk plots showed that the Michaelis–Menten constant (Km) remained nearly unchanged in the presence of granschisandrin (1) compared with the control (0.65 vs 0.55 mM), whereas the maximum reaction velocity (Vmax) decreased from 31.68 μM/min in the control to 12.98 μM/min in the presence of the compound, using different concentrations of L-tyrosine as the substrate. These results indicate that granschisandrin (1) acts as a predominantly non-competitive inhibitor, suggesting that it binds to an allosteric site rather than to the catalytic site of the enzyme.

## Conclusions

This work presents a detailed analysis of lignans in *S. chinensis* fruits, a plant known for its neuroprotective effects. 25 isolated lignans were first quantified and then tested against the tyrosinase enzyme. The spectrophotometric assay revealed that, among all the isolated lignans, FR and DB types of lignans showed better inhibition than DCO and AT types. The results indicated that granschisandrin (1), a furan lignan, showed the best inhibition with an IC_50_ value of 142.75 µM.

The reduced dataset (rd-STD NMR) approach allowed us to determine the binding epitope map of granschisandrin, revealing that the regions involved in binding with the tyrosinase protein are the aromatic protons along with the methyl group at C-7. Subsequently, STD NMR competition experiments between granschisandrin and kojic acid supported the binding of granschisandrin (1) to an allosteric pocket located near the catalytic site of the enzyme. This result was corroborated by *in silico* studies, rationalizing that the preferential binding site of compound 1 appears to be near the catalytic site of the enzyme, where kojic acid is known to bind. Kinetic analysis demonstrated that granschisandrin (1) exhibits a predominantly non-competitive inhibition pattern, as evidenced by changes in Vmax with minimal effects on Km, supporting its interaction with a site distinct from the catalytic active site. In literature, it is reported that many lignans, including those from sources such as *Schisandra chinensis*, are known to have lipophilic characteristics that allow them to cross the blood-brain barrier, a key factor in their potential neuroprotective effects (Das and Devi, 2019; Wu et al., 2016; Zhang et al., 2020). Based on this consideration, the obtained results encourage the potential use of *S. chinesis* lignans in the treatment and prevention of diseases related to overexpression of the enzyme tyrosinase. Accordingly, further cellular, pharmacokinetic, and *in vivo* studies are necessary to confirm these preliminary findings, with reference to potential neurodegenerative diseases where the accumulation of neuromelanin represents a determining factor and in line with the traditional medicinal use of the plant.

## Data Availability

The original contributions presented in the study are included in the article/**Supplementary Material**. Further inquiries can be directed to the corresponding authors.
