# Supplementary material for: Lignans in Schisandra chinensis green extracts: quantitative analysis and evaluation of tyrosinase inhibitory activity through spectrophotometric assay, in silico studies, and STD NMR spectroscopy
Source: Front Plant Sci. 2026 Jul 8;17:1844111. doi: 10.3389/fpls.2026.1844111 (PMC13388266; doi:10.3389/fpls.2026.1844111)
Supplement: Supplementary file 1 [file DataSheet1.docx]

Supplementary Material

# Supplementary Figures and Tables

**Supplementary Figure 1**. ^1^H NMR spectrum of compound **1**

**Supplementary Figure 2**. HSQC spectrum of compound **1**

**Supplementary Figure 3**. HMBC spectrum of compound **1**

**Supplementary Figure 4**. ROESY spectrum of compound **1**

**Supplementary Figure 5**. STD NMR spectrum of 1 mM compound **1** in presence of 16.5 µM of tyrosinase.

**Supplementary Figure 6**. STD NMR spectrum of 1 mM compound **1** in presence of 16.5 µM of tyrosinase and **1** mM of kojic acid

**Supplementary Table 1.** Fractions of *S. chinensis* purified using HPLC and isolated lignans.

**Supplementary Table 2.** LC–MS/MS conditions for quantitative analysis of compounds **1**-**25**, by positive ion MRM mode

**Supplementary Table 3.** LC–MS/MS regression line, LOD, LOQ for quantitative analysis of compounds **1**-**25**, by positive ion MRM mode.

**Supplementary Table 4.** Quantitative results of furane (FR) lignans (**1**, **2** ,**4**) expressed as mg/g of extract ± SD in green extracts of *S. chinensis*

**Supplementary Table 5.** Quantitative results of dibenzylbutane (DB) lignans (**5**, **6** ,**8**, **9**, **11**) expressed as mg/g of extract ± SD in green extracts of *S. chinensis*

**Supplementary Table 6.** Quantitative results of dibenzocyclooctadiene (DCO) lignans (**15**-**22**, **25**) expressed as mg/g of extract ± SD in green extracts of *S. chinensis*

**Supplementary Table 7.** Quantitative results of aryltetraline (AT) lignans (**14**) expressed as mg/g of extract ± SD in green extracts of *S. chinensis*

**Supplementary Table 8.** Tyrosinase inhibition activity results of *S. chinensis* isolated lignans

**Supplementary Table 9.** STD NMR integration values at short (0.75 s) and long (6 s) saturation times, k_sat_, STD_0_ and the relative STD percentage (epitope) of all the protons of Granschisandrin (**1**) for its binding to the tyrosinase enzyme.

**Supplementary Figure 7.** Superposition of the docked binding mode of tropolone with its experimental crystallographic pose in the tyrosinase crystal structure.

**Supplementary Figure 8.** Protein - RMSF plot of the tyrosinase in complex with kojic acid.

**Supplementary Figure 9.** Protein - RMSF plot of the allosteric tyrosinase site in complex with compound **1**.

**Supplementary Figure 10.** Tyrosinase – compound **1** interactions monitored throughout the molecular dynamics simulation, considering compound **1** in the catalytic site of the protein as the starting point.

**Supplementary Figure 11**. A) Tyrosinase – kojic acid interactions in the ternary complex, monitored throughout the molecular dynamics simulation. B) Tyrosinase – compound **1** interactions in the ternary complex, monitored throughout the molecular dynamics simulation.

**Supplementary Figure 12.** Protein - RMSF plot of the tyrosinase in complex with kojic acid and compound **1**.

**Supplementary Figure 13**. RMSD plot of the tyrosinase in complex with kojic acid and compound **1**.

**Supplementary Figure 14.** Lineweaver–Burk plots for tyrosinase inhibition by compound **1**


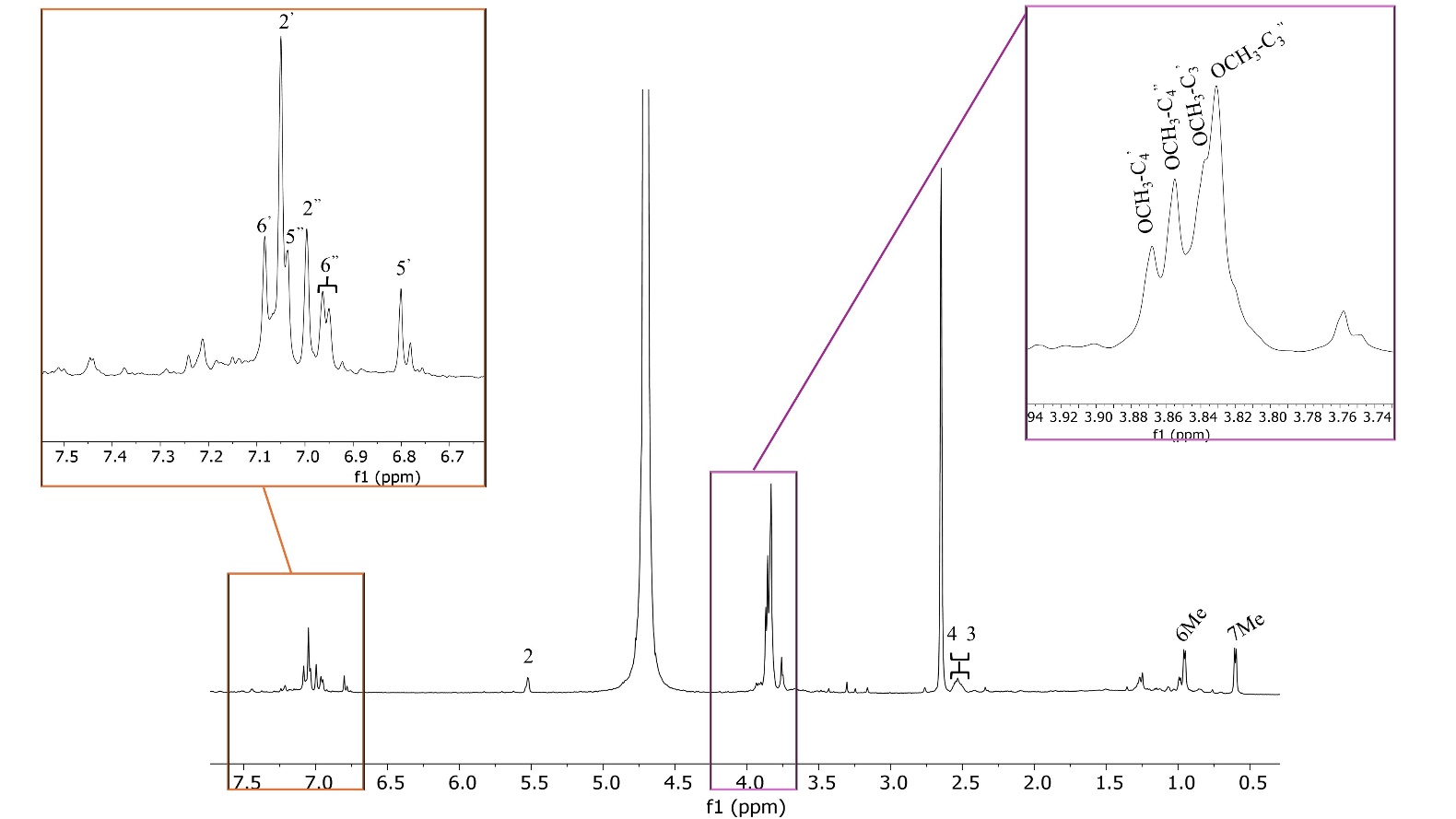


**Supplementary Figure 1.** ^1^H NMR spectrum of compound **1** acquired in D_2_O 5% of DMSO

**
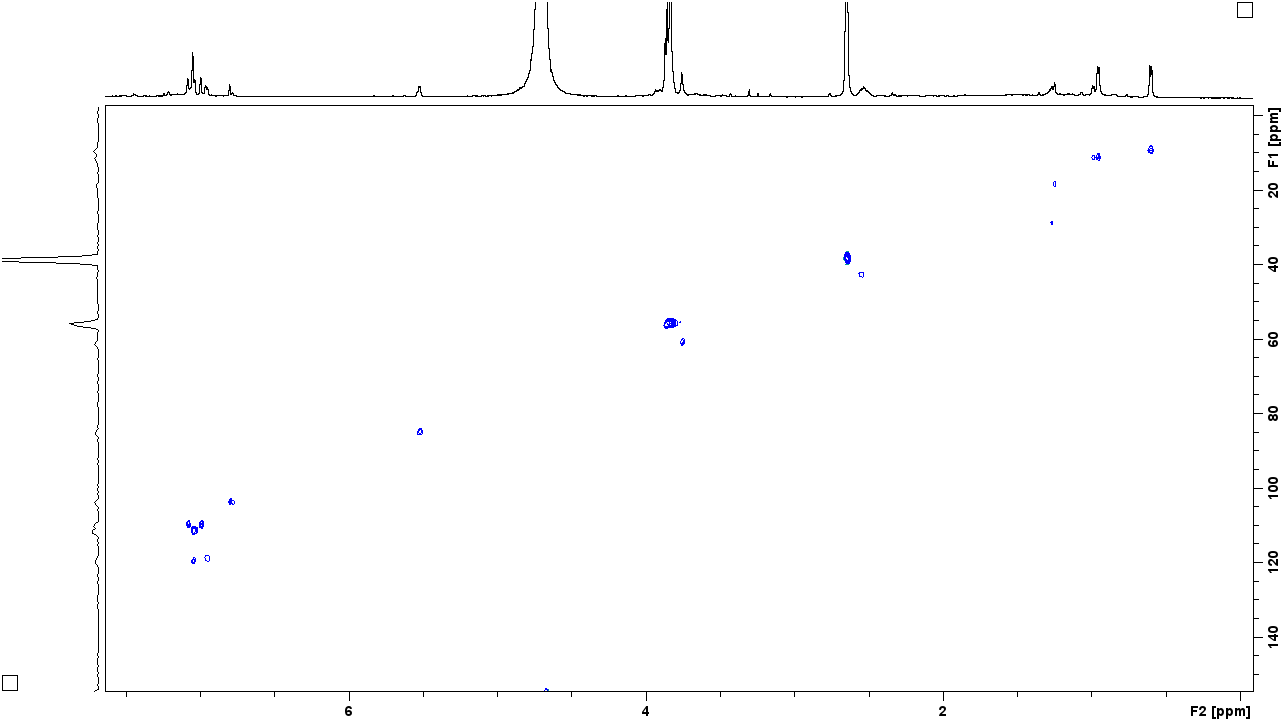
**

**Supplementary Figure 2.** HSQC spectrum of compound **1** acquired in D_2_O 5% of DMSO

**
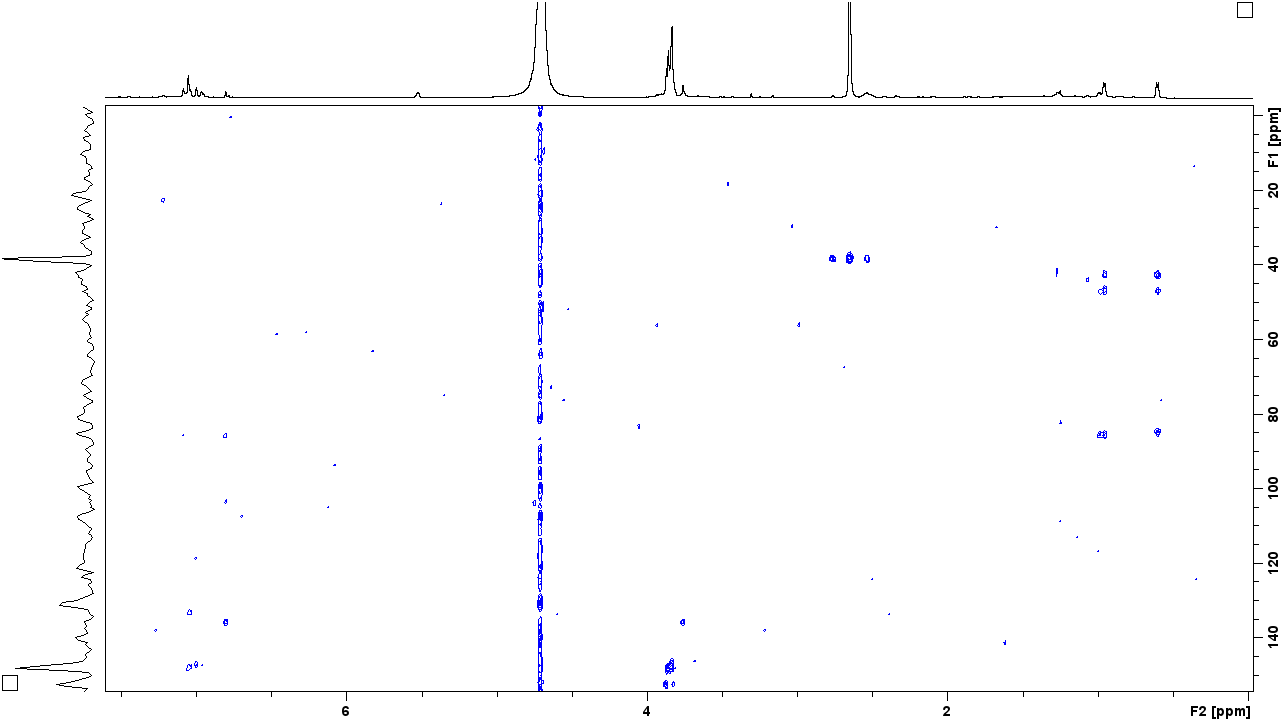
**

**Supplementary Figure 3.** HMBC spectrum of compound **1** acquired in D_2_O 5% of DMSO


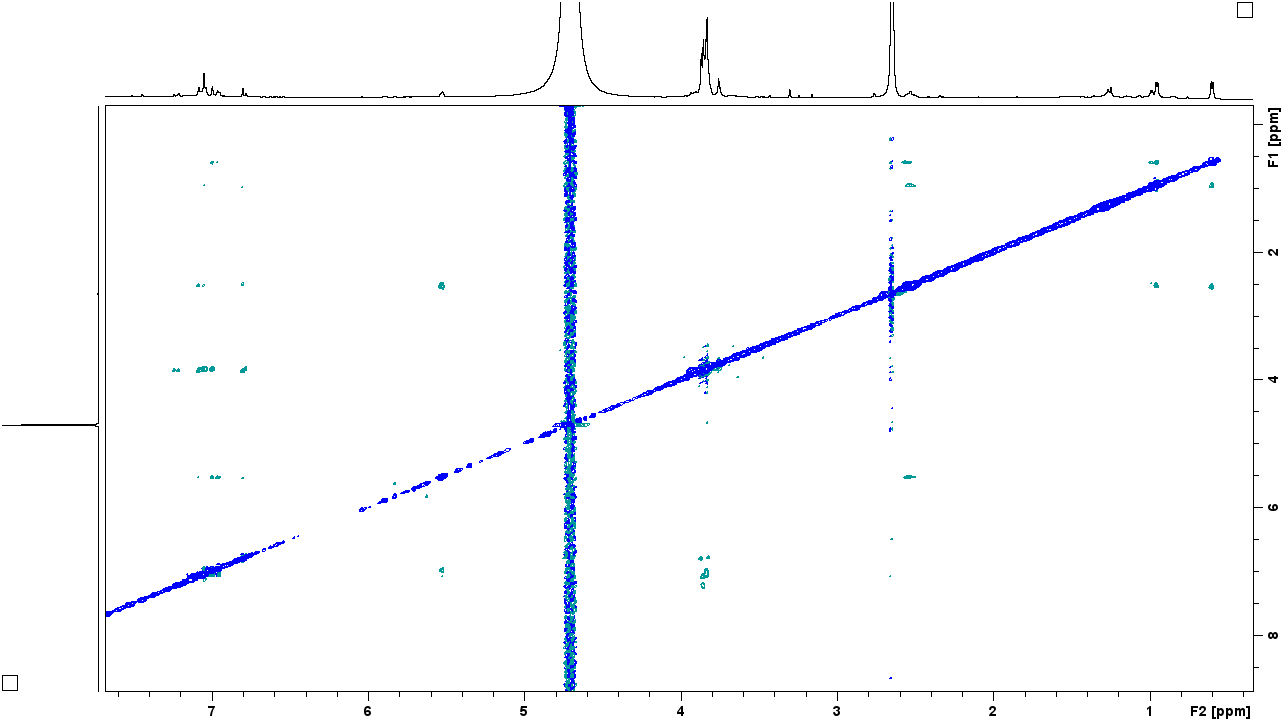


**Supplementary Figure 4.** ROESY spectrum of compound **1**

**
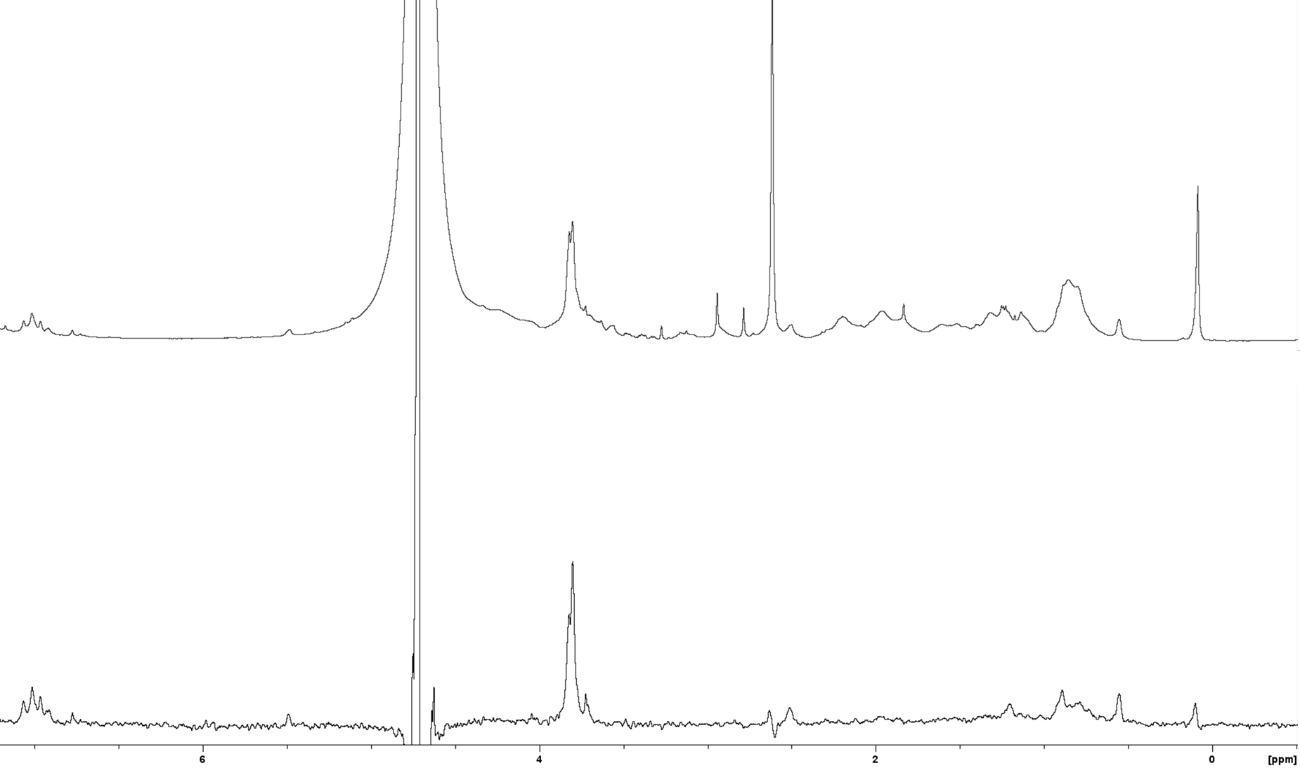
**

**Supplementary Figure 5.** STD NMR spectrum of compound **1** (1mM) in presence of tyrosinase (16.5 µM) (Top: off resonance spectrum with irradiation frequency at 40 ppm. Bottom: on resonance spectrum with irradiation frequency at -1 ppm; scale 1:10).

**
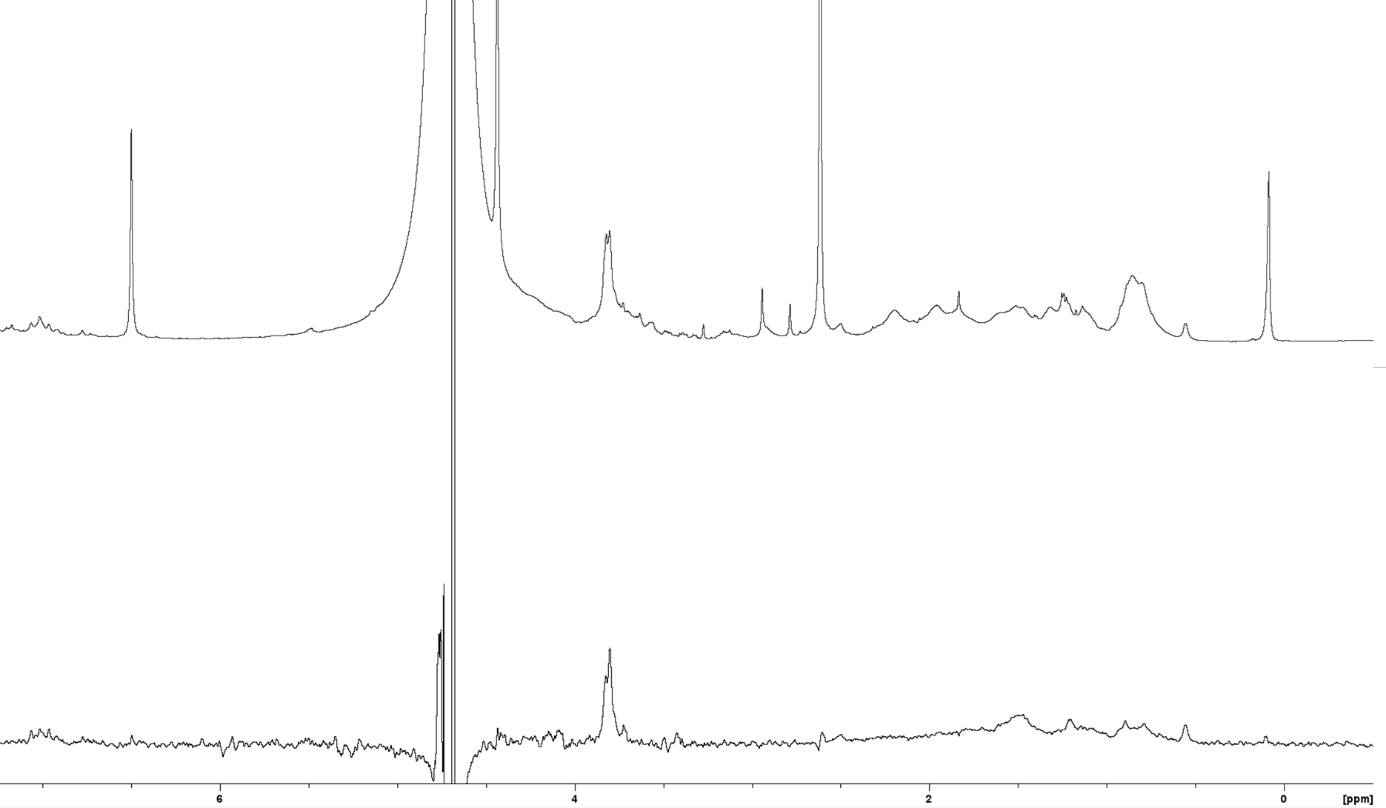
**

**Supplementary Figure 6.** STD NMR spectrum of compound **1** (1mM) in presence of tyrosinase **(**16.5 µM) and kojic acid (1 mM). (Top: off resonance spectrum with irradiation frequency at 40 ppm. Bottom: on resonance spectrum with irradiation frequency at -1 ppm; scale 1:10).

**Supplementary Table 1.** Fractions of *S. chinensis* purified using HPLC and isolated lignans.

|  | Fractions 14–17  (619.7 mg) | Fractions 18–19  (712.9 mg) | Fractions 20–22  (693.2 mg) | Fractions 23–25  (88.7 mg) | Fractions 26–32  (82.2 mg) |
| --- | --- | --- | --- | --- | --- |
| Isolated lignan | **15** | **17** | **14** | **6** | **12** |
| Weight (mg) | 0.3 | 0.8 | 0.9 | 1.6 | 0.2 |
| Rt (min) | 32.1 | 33.2 | 31.0 | 35.6 | 41.4 |
|  |  |  |  |  |  |
| Isolated lignan | **19** | **23** | **7** | **2** |  |
| Weight (mg) | 0.3 | 0.4 | 0.5 | 0.6 |  |
| Rt (min) | 37.6 | 16.3 | 39.3 | 36.6 |  |
|  |  |  |  |  |  |
| Isolated lignan | **16** | **11** | **9** | **8** |  |
| Weight (mg) | 0.6 | 0.5 | 0.4 | 0.9 |  |
| Rt (min) | 6.9 | 41.9 | 41.4 | 40.6 |  |
|  |  |  |  |  |  |
| Isolated lignan | **20** | **21** | **1** | **13** |  |
| Weight (mg) | 0.4 | 0.5 | 1.2 | 0.2 |  |
| Rt (min) | 37.9 | 44.5 | 37.3 | 20.1 |  |
|  |  |  |  |  |  |
| Isolated lignan | **18** | **22** | **4** | **10** |  |
| Weight (mg) | 0.4 | 0.2 | 0.5 | 0.7 |  |
| Rt (min) | 42.6 | 44.8 | 38.6 | 45.4 |  |
|  |  |  |  |  |  |
| Isolated lignan | 5 |  | **3** | **24** |  |
| Weight (mg) | 0.5 |  | 0.3 | 0.8 |  |
| Rt (min) | 44.6 |  | 38.9 | 37.8 |  |
|  |  |  |  |  |  |
| Isolated lignan |  |  |  | **25** |  |
| Weight (mg) |  |  |  | 0.3 |  |
| Rt (min) |  |  |  | 44.3 |  |

**Supplementary Table 2.** LC–MS/MS conditions for quantitative analysis of compounds **1**-**25**, by positive ion MRM mode.

| **Compounds** | **MRM transition** | DP | CE | EP | CXP |
| --- | --- | --- | --- | --- | --- |
| **1** | 373 -> 235 | 27.90 | 20.00 | 8.90 | 29.60 |
| **2** | 343 -> 219 | 27.90 | 20.00 | 8.90 | 29.60 |
| **3** | 409 -> 289 | 27.90 | 20.00 | 8.90 | 29.60 |
| **4** | 357 -> 235 | 27.90 | 20.00 | 8.90 | 29.60 |
| **5** | 399 -> 369 | 33.60 | 30.00 | 6.30 | 11.50 |
| **6** | 353 -> 203 | 33.60 | 30.00 | 6.30 | 11.50 |
| **7** | 271 -> 255 | 32.00 | 35.00 | 8.00 | 11.50 |
| **8** | 371 -> 205 | 33.60 | 30.00 | 6.30 | 11.50 |
| **9** | 391 -> 237 | 33.60 | 30.00 | 6.30 | 11.50 |
| **10** | 351-> 203 | 33.60 | 30.00 | 6.30 | 11.50 |
| **11** | 359 -> 221 | 33.60 | 30.00 | 6.30 | 11.50 |
| **12** | 363 -> 311 | 42.30 | 30.00 | 13.00 | 19.80 |
| **13** | 341-> 219 | 42.30 | 30.00 | 13.00 | 19.80 |
| **14** | 379 -> 311 | 42.30 | 30.00 | 13.00 | 19.80 |
| **15** | 388-> 301 | 30.86 | 29.52 | 10.86 | 50.00 |
| **16** | 405-> 301 | 30.86 | 29.52 | 10.86 | 50.00 |
| **17** | 455-> 361 | 30.86 | 29.52 | 10.86 | 50.00 |
| **18** | 417 -> 301 | 30.86 | 29.52 | 10.86 | 50.00 |
| **19** | 559 -> 415 | 30.86 | 29.52 | 10.86 | 50.00 |
| **20** | 537 -> 437 | 30.86 | 35.00 | 10.86 | 50.00 |
| **21** | 543 -> 399 | 30.86 | 29.52 | 10.86 | 50.00 |
| **22** | 521 -> 399 | 30.86 | 29.52 | 10.86 | 50.00 |
| **23** | 415 -> 347 | 30.86 | 29.52 | 10.86 | 50.00 |
| **24** | 543 -> 421 | 30.86 | 29.52 | 10.86 | 50.00 |
| **25** | 527 -> 321 | 30.86 | 35.00 | 10.86 | 50.00 |

DP, Declustering Potential; CE, Collision energy; EP, Entrance potential; CXP, Collision Cell Exit Potential.

**Supplementary Table 3.** LC–MS/MS regression line, LOD, LOQ for quantitative analysis of compounds **1**-**25**, by positive ion MRM mode.

| **Compounds** | **R^2^** | **Regression line** | LOD  mg/L | LOQ  mg/L |
| --- | --- | --- | --- | --- |
| **1** | 0.992 | y = 0.0142x-0.1596 | 0.0114 | 0.0379 |
| **2** | 0.999 | y = 0.0009x+0.0003 | 0.0188 | 0.0628 |
| **3** | 0.996 | y = 0.0126x+0.0258 | 0.0070 | 0.0235 |
| **4** | 0.994 | y = 0.0138x+0.0321 | 0.053 | 0.0175 |
| **5** | 0.996 | y= 0.0108x+ 0.361 | 0.0020 | 0.0068 |
| **6** | 0.994 | y = 9.96^e-6^x+0.0155 | 0.0061 | 0.0201 |
| **7** | 0.990 | y = 0.0122x-0.3730 | 0.0093 | 0.0310 |
| **8** | 0.999 | y = 0.0087x+0.0002 | 0.0032 | 0.0107 |
| **9** | 0.997 | y = 0.0087x+0.0018 | 0.0012 | 0.0042 |
| **10** | 0.994 | y= 0.00651x+ 0.4534 | 0.0023 | 0.0077 |
| **11** | 0.990 | y= 0.0164x+ 0.816 | 0.0017 | 0.0058 |
| **12** | 0.997 | y= 0.0025x+ 0.040 | 0.0037 | 0.0123 |
| **13** | 0.999 | y= 0.0024x+ 0.0367 | 0.0061 | 0.0204 |
| **14** | 0.996 | y= 0.0015x+ 0.0138 | 0.0027 | 0.0091 |
| **15** | 0.997 | y = 0.0005x+0.0005 | 0.0007 | 0.0023 |
| **16** | 0.997 | y = 0.0012x+0.0006 | 0.0082 | 0.0820 |
| **17** | 0.997 | y = 0.0003x+0.0003 | 0.0011 | 0.0036 |
| **18** | 0.996 | y = 0.0004x+0.0005 | 0.0003 | 0.0010 |
| **19** | 0.999 | y= 0.0005+ 0.006 | 0.0017 | 0.0056 |
| **20** | 0.999 | y = 0.0007x+0.0223 | 0.0022 | 0.0074 |
| **21** | 0.992 | y = 6.26^e-6^x+0.00184 | 0.0050 | 0.0167 |
| **22** | 0.999 | y= 0.0011x+ 0.0218 | 0.0029 | 0.0098 |
| **23** | 0.998 | y= 0.0003x+ 0.0087 | 0.0009 | 0.0030 |
| **24** | 0.999 | y= 0.0001x+ 0.0062 | 0.0012 | 0.0041 |
| **25** | 0.999 | y= 0.0002x+ 0.0061 | 0.0018 | 0.0060 |

LOQ, limit of quantification; LOD, limit of detection; LOD and LOQ expressed as mg/L.

**Supplementary Table 4.** Quantitative results of furane (FR) lignans (**1**, **2** ,**4**) expressed as mg/g of extract ± SD in green extracts of *S. chinensis*

| FR (mg/g of extract) | **1** | **2** | **4** |
| --- | --- | --- | --- |
| SLDE_50 | 1.250 ± 0.152 | 0.221± 0.044 | 0.096± 0.022 |
| SLDE_75 | 2.394 ± 0.219 | 1.514± 0.154 | 0.292± 0.037 |
| SLDE_100 | 5.147 ± 0.319 | 4.673± 0.155 | 1.131± 0.039 |
| UAE_50 | 1.414 ± 0.124 | 0.079± 0.027 | 0.353± 0.012 |
| UAE_75 | 2.340 ± 0.183 | 0.092± 0.007 | 0.221± 0.018 |
| UAE_100 | 1.972± 0.204 | 2.760± 0.248 | 0.365± 0.039 |

**Supplementary Table 5.** Quantitative results of dibenzylbutane (DB) lignans (**5**, **6** ,**8**, **9**, **11**) expressed as mg/g of extract ± SD in green extracts of *S. chinensis*

| DB (mg/g of extract) | **5** | **6** | **8** | **9** | **11** |
| --- | --- | --- | --- | --- | --- |
| SLDE_50 | 0.649± 0.037 | 1.050± 0.125 | 0.567±0.114 | 0.077± 0.019 | 0.726± 0.053 |
| SLDE_75 | 4.303± 0.255 | 1.946± 0.223 | 3.933±0.245 | 0.131± 0.019 | 1.627± 0.156 |
| SLDE_100 | 7.626± 0.280 | 4.866± 0.244 | 6.876±0.279 | 0.194± 0.021 | 4.453± 0.180 |
| UAE_50 | 0.280± 0.025 | 1.112± 0.158 | 0.167± 0.023 | 0.067± 0.001 | 1.032± 0.097 |
| UAE_75 | 0.193± 0.008 | 2.055± 0.166 | 0.145± 0.012 | 0.064± 0.009 | 1.859± 0.136 |
| UAE_100 | 0.976± 0.065 | 1.762± 0.298 | 0.892± 0.332 | 0.091± 0.010 | 2.383± 0.050 |

**Supplementary Table 6.** Quantitative results of dibenzocyclooctadiene (DCO) lignans (**15**-**22**, **25**) expressed as mg/g of extract ± SD in green extracts of *S. chinensis*

| DCO (mg/g of extract) | **15** | **16** | **17** | **18** | **19** | **20** | **21** | **22** | **25** |
| --- | --- | --- | --- | --- | --- | --- | --- | --- | --- |
| SLDE_50 | 0.084± 0.001 | 0.155± 0.014 | 0.055± 0.014 | 0.184± 0.011 | 0.859± 0.075 | 0.174± 0.023 | 0.343± 0.074 | 0.363± 0.019 | 0.160± 0.011 |
| SLDE_75 | 0.945± 0.076 | 1.498±0.098 | 0.504± 0.037 | 1.829± 0.064 | 3.894± 0.226 | 1.796± 0.134 | 5.480± 0.246 | 2.487± 0.042 | 0.698± 0.074 |
| SLDE_100 | 1.677± 0.167 | 2.643± 0.158 | 1.889± 0.037 | 3.100± 0.175 | 6.031± 0.325 | 2.897± 0.168 | 6.467± 0.235 | 2.873± 0.224 | 1.483± 0.015 |
| UAE_50 | 0.008± 0.001 | 0.013± 0.003 | 0.425± 0.028 | 0.019± 0.001 | 0.788± 0.046 | 0.016± 0.001 | 0.625± 0.136 | 0.198± 0.016 | 0.283± 0.042 |
| UAE_75 | 0.006± 0.002 | 0.091± 0.002 | 0.434± 0.018 | 0.017± 0.002 | 0.240± 0.025 | 0.014± 0.001 | 0.119± 0.019 | 0.118± 0.006 | 0.449± 0.021 |
| UAE_100 | 0.083± 0.051 | 0.105± 0.018 | 0.856± 0.016 | 0.176± 0.011 | 1.276± 0.116 | 0.131± 0.022 | 1.559± 0.153 | 0.499± 0.035 | 0.785± 0.081 |

**Supplementary Table 7.** Quantitative results of aryltetraline (AT) lignans (**14**) expressed as mg/g of extract ± SD in green extracts of *S. chinensis*

| AT (mg/g of extract) | **14** |
| --- | --- |
| SLDE_50 | 0.018± 0.006 |
| SLDE_75 | 0.014± 0.006 |
| SLDE_100 | 0.036± 0.001 |
| UAE_50 | 0.021± 0.001 |
| UAE_75 | 0.021± 0.002 |
| UAE_100 | 0.011± 0.004 |

**Supplementary Table 8.** Tyrosinase inhibition activity of *S. chinensis* isolated lignans

| **Isolated lignans** | **IC_50_ (µM) ±SD** | **RA** |
| --- | --- | --- |
| **1** | 142.75±4.44 | 1.43 |
| **2** | 245.16±4.00 | 2.45 |
| **3** | 248.51±4.98 | 2.48 |
| **4** | 213.00±14.82 | 2.13 |
| **5** | 184.74±5.13 | 1.85 |
| **6** | 170.64±6.06 | 1.71 |
| **7** | 215.71±9.98 | 2.16 |
| **8** | 217.56±11.91 | 2.17 |
| **9** | 177.64±12.46 | 1.78 |
| **10** | 234.08±6.82 | 2.34 |
| **11** | 210.42±6.75 | 2.10 |
| **12** | 326.52±10.60 | 3.26 |
| **13** | 332.15±3.96 | 3.32 |
| **14** | 335.32±4.68 | 3.35 |
| **15** | 170.15±8.70 | 1.70 |
| **16** | 213.23±5.77 | 2.13 |
| **17** | 237.28±5.02 | 2.37 |
| **18** | 246.34±6.12 | 2.46 |
| **19** | 193.72±4.50 | 1.94 |
| **20** | 224.57±5.79 | 2.24 |
| **21** | 270.45±6.38 | 2.70 |
| **22** | 181.86±4.97 | 1.82 |
| **23** | 253.88±4.72 | 2.54 |
| **24** | 188.33±5.23 | 1.88 |
| **25** | 189.06±7.25 | 1.89 |
| **K.A.** | 100.07±4.50 |  |

**Supplementary Table 9.** STD NMR integration values at short (0.75 s) and long (6 s) saturation times, k_sat_, STD_0_ and the relative STD percentage (epitope) of all the protons of Granschisandrin (**1**) for its binding to the tyrosinase enzyme.

| **Proton name** | **STD at short saturation time** | **STD at long saturation time** | | **k_sat_** | **STD_0_** | | **Epitope (%)** |
| --- | --- | --- | --- | --- | --- | --- | --- |
| 6'ar | 11.24 | 18.85 | | 1.21 | 22.80 | | 87.69 |
| 2'ar | 9.61 | 13.53 | | 1.65 | 22.35 | | 85.96 |
| 5''ar | 10.19 | 15.66 | | 1.40 | 21.96 | | 84.48 |
| 2''ar | 12.39 | 20.78 | | 1.21 | 25.13 | | 96.66 |
| 6''ar | 11.8 | 22.25 | 1.08 | 22.42 | 86.24 |  |  |
| 5'ar | 12.15 | 18.85 | | 1.38 | 25.00 | | 100.00 |
| OCH_3_-C4' | 6.24 | 11.46 | | 1.05 | 12.01 | | 46.22 |
| OCH_3_-C4'' | 6.06 | 9.06 | | 1.47 | 13.35 | | 51.36 |
| OCH_3_-C3'' | 8.04 | 15.81 | | 0.95 | 14.97 | | 57.60 |
| 4H | 6.96 | 9.33 | | 1.83 | 17.05 | | 65.57 |
| 3H | 7.17 | 9.8 | | 1.75 | 17.19 | | 66.11 |
| 7Me | 9.31 | 12.51 | | 1.82 | 22.74 | | 87.47 |

**
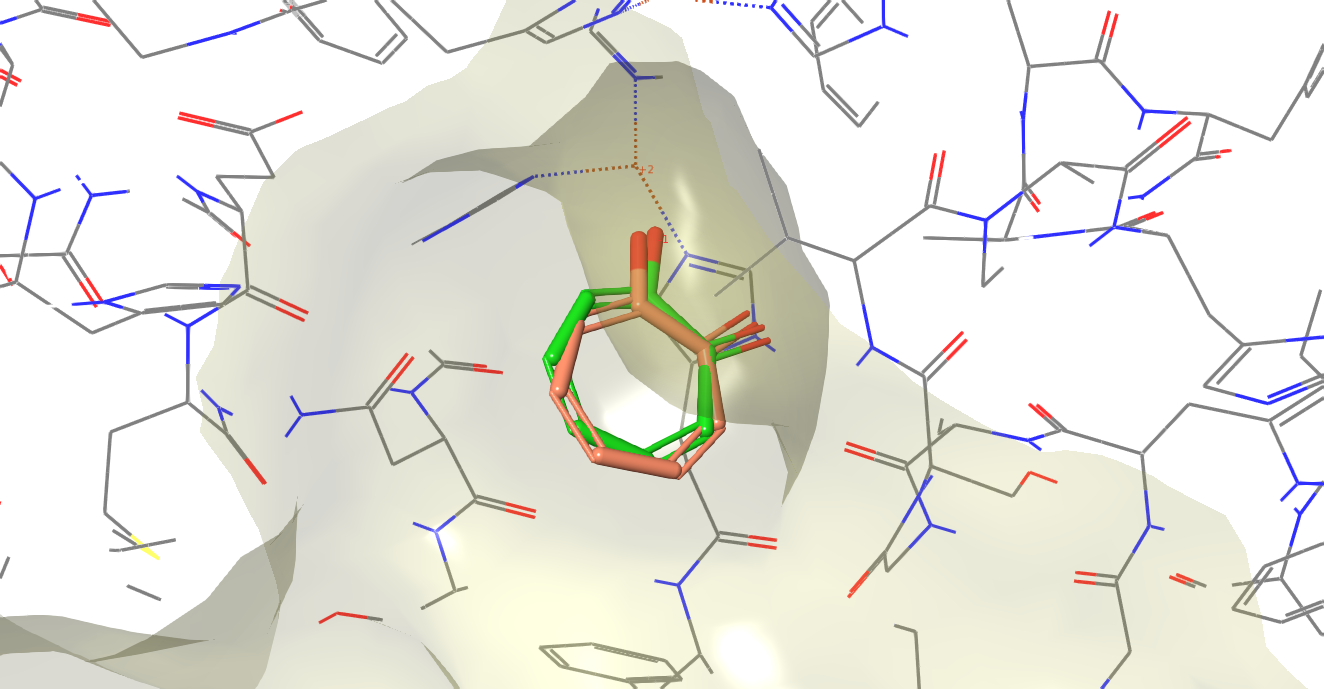
**

**Supplementary Figure 7.** Superposition of the docked binding mode of tropolone (atoms colored by type: carbon in orange, oxygen in red) with its experimental crystallographic pose (atoms colored by type: carbon in green, oxygen in red) in the tyrosinase crystal structure (PDB code: 2Y9X).


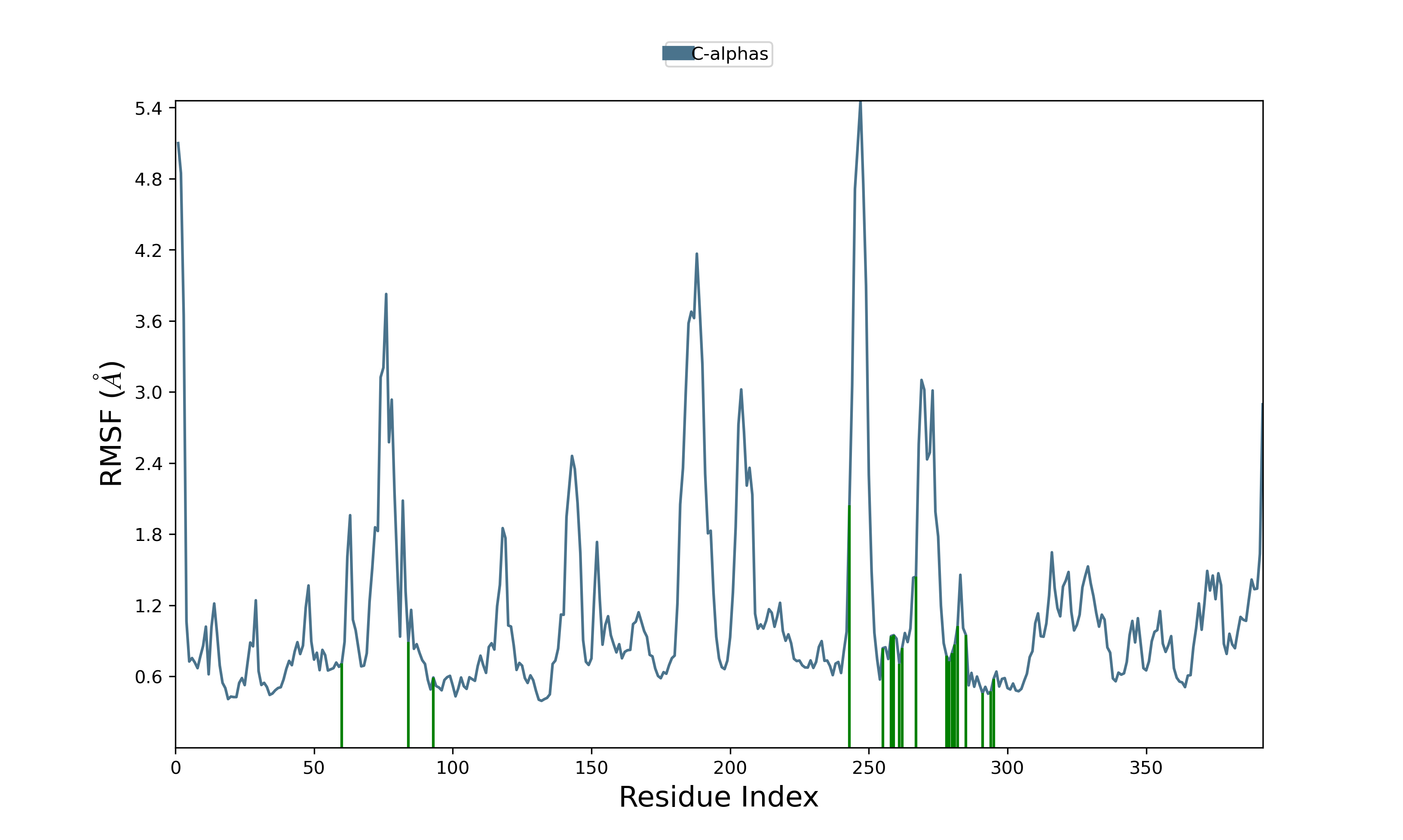


**Supplementary Figure 8.** Protein - RMSF plot of the tyrosinase in complex with kojic acid. Peaks indicate areas of the protein that fluctuate during the simulation, according to the related RMSF values (Å). Protein residues that interact with the ligand are marked with green-colored vertical bars.

**
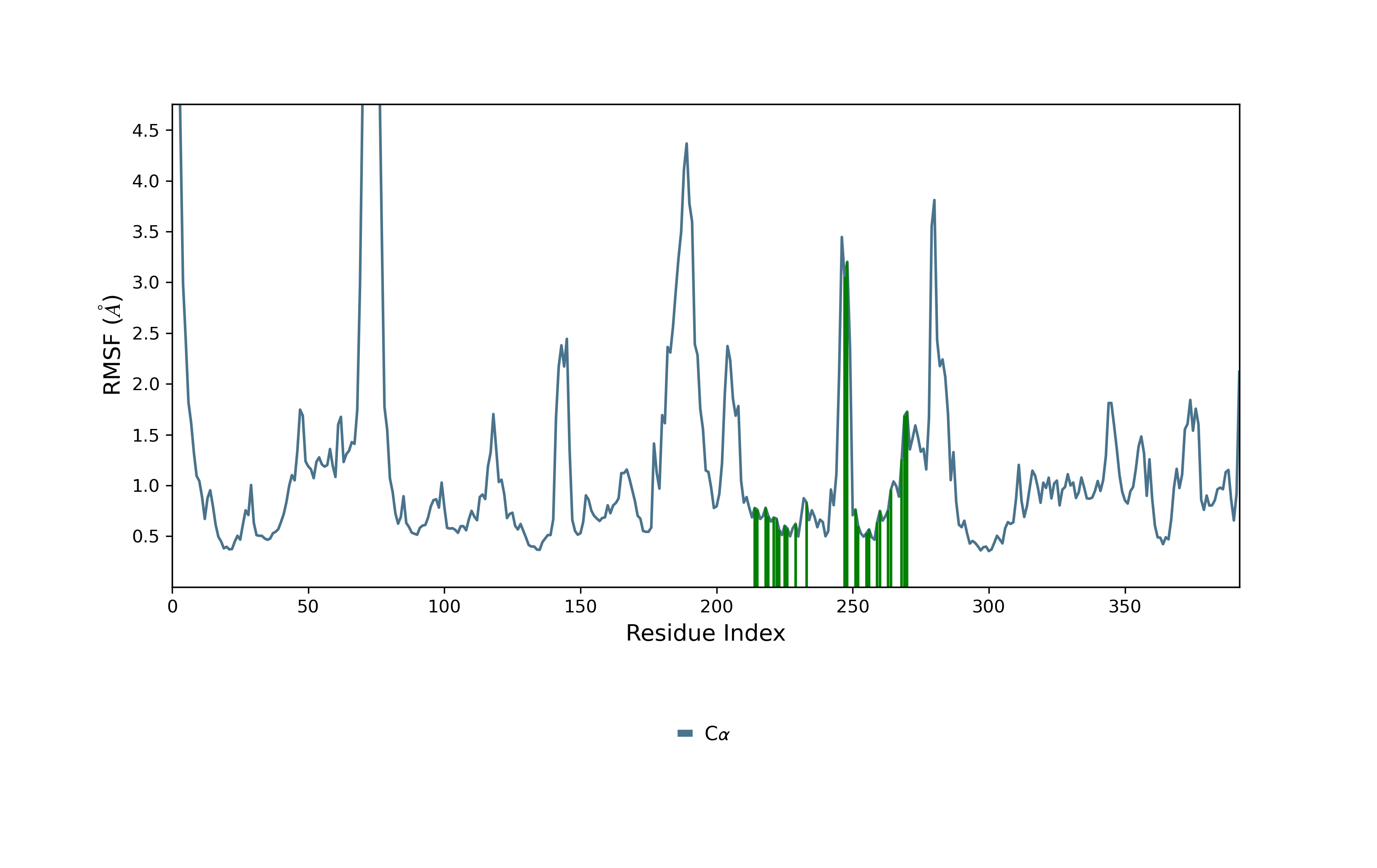
**

**Supplementary Figure 9.** Protein - RMSF plot of the allosteric tyrosinase site in complex with compound **1**. Peaks indicate areas of the protein that fluctuate during the simulation, according to the related RMSF values (Å). Protein residues that interact with the ligand are marked with green-colored vertical bars.

**
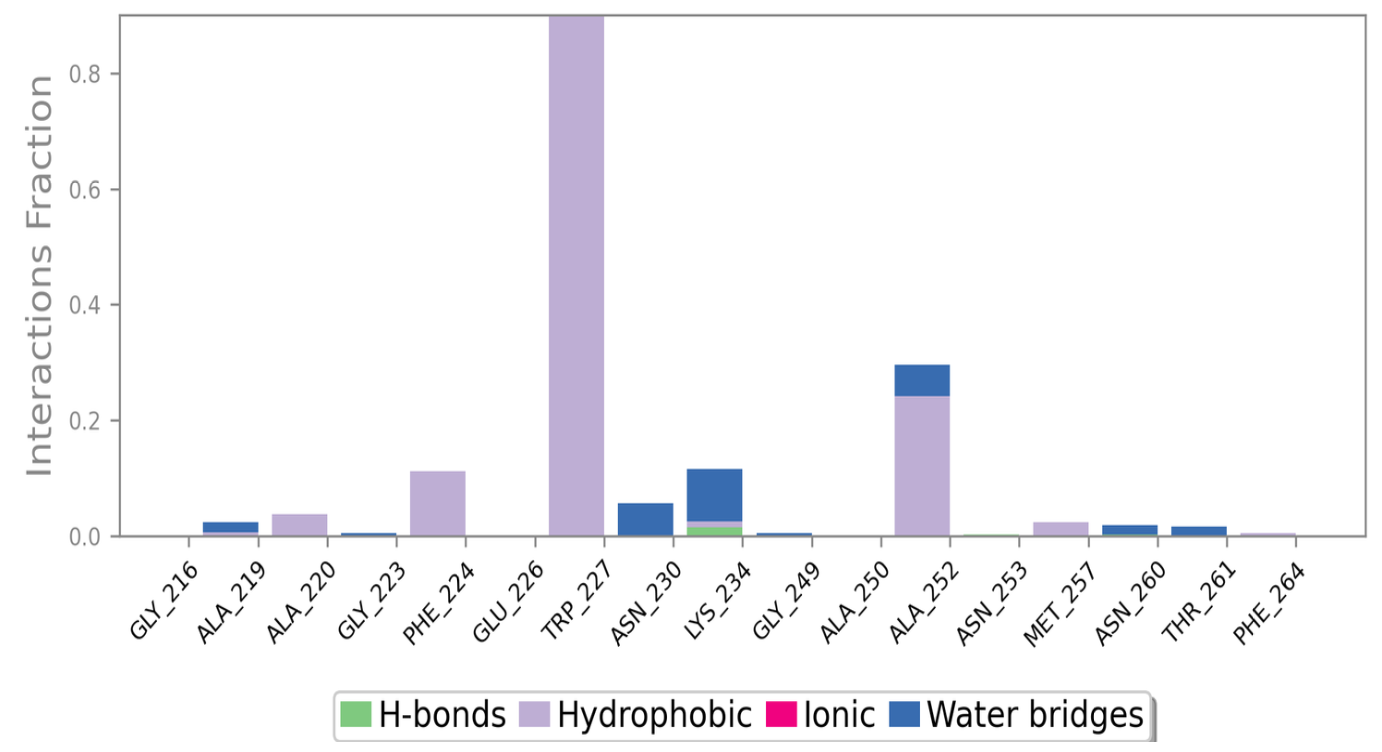
**

**Supplementary Figure 10.** Tyrosinase – compound **1** interactions monitored throughout the molecular dynamics simulation, considering compound **1** in the catalytic site of the protein as the starting point (values over 1.0 are possible as some protein residue may make multiple contacts of the same subtype with the ligand).

**
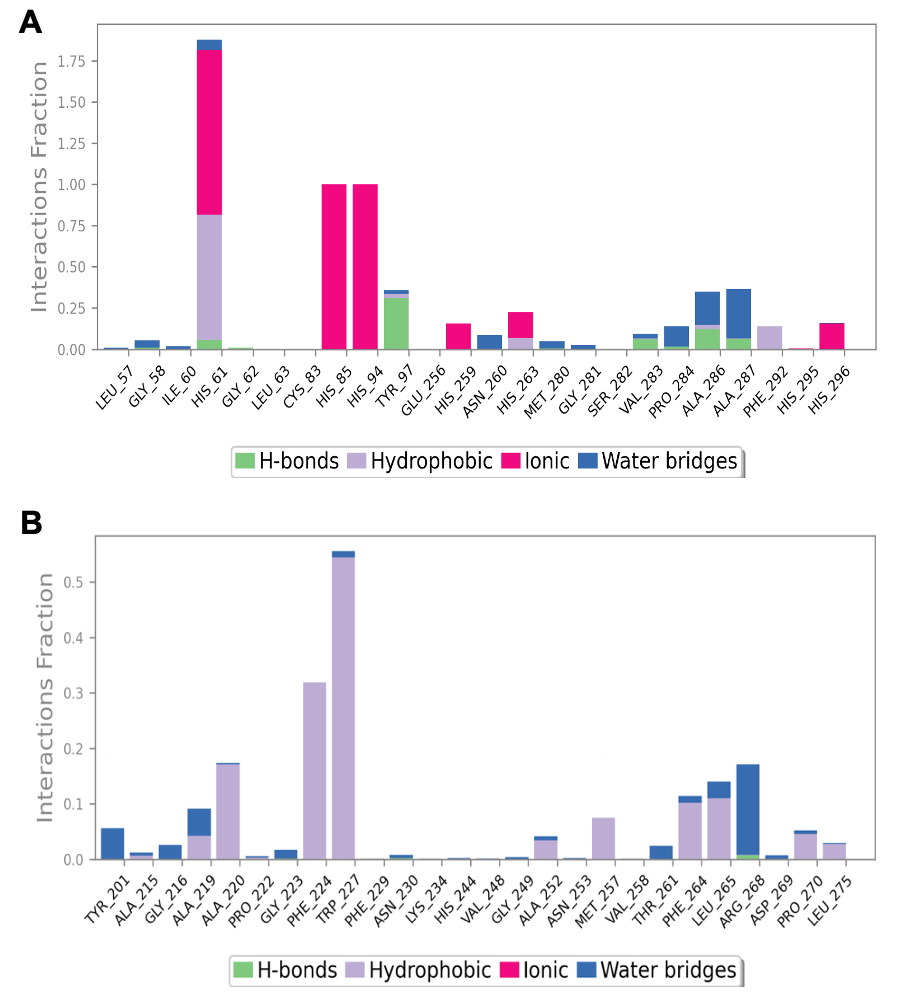
**

**Supplementary Figure 11**. A) Tyrosinase – kojic acid interactions in the ternary complex, monitored throughout the molecular dynamics simulation. B) Tyrosinase – compound **1** interactions in the ternary complex, monitored throughout the molecular dynamics simulation. Values over 1.0 are possible as some protein residue may make multiple contacts of the same subtype with the ligand.


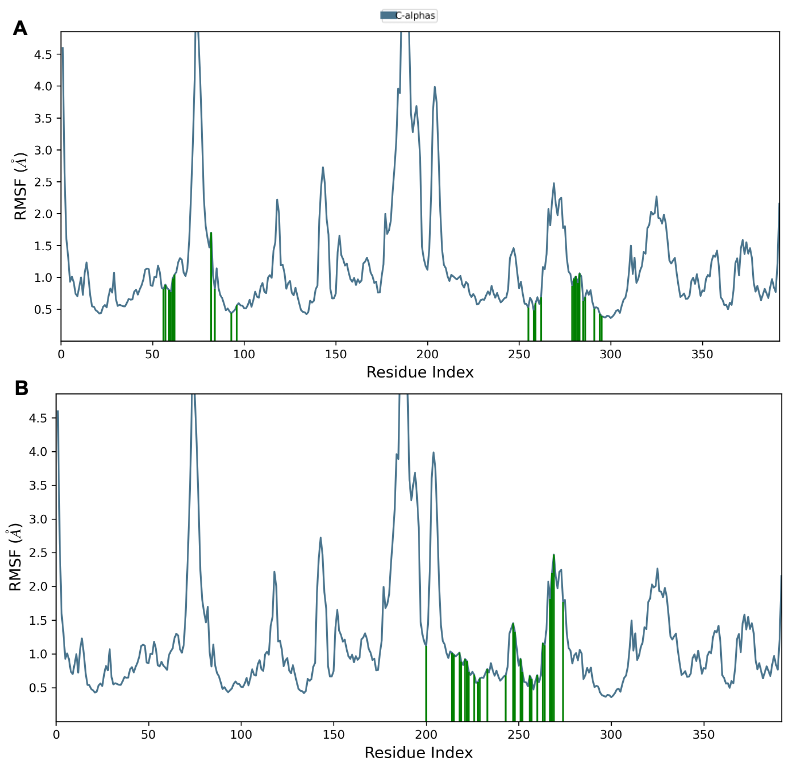


**Supplementary Figure 12.** Protein - RMSF plot of the tyrosinase in complex with kojic acid and compound **1**. Green vertical bars indicate the protein residues interacting with A) the kojic acid and B) compound **1**.

**Supplementary Figure 13**. RMSD plot of the tyrosinase in complex with kojic acid and compound **1**. The trajectory frame was extracted every 2000 ps and superimposed onto the initial structure (frame 0). RMSD was calculated for backbone atoms of the protein and all heavy atoms of the ligand.


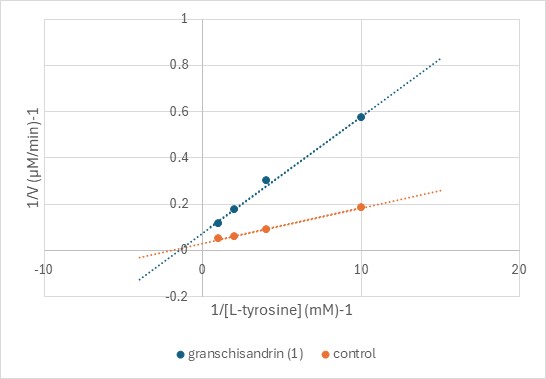


**Supplementary Figure 14.** Lineweaver–Burk plots for tyrosinase inhibition in the presence of Compound 1 (blue lines) at different concentrations of the substrate L-tyrosine (0.10, 0.25, 0.50, and 1.00 mM), compared with the control without Compound **1** (orange lines) at the same L-tyrosine concentrations.
